# Hedging against Antiviral Resistance during the Next Influenza Pandemic Using Small Stockpiles of an Alternative Chemotherapy

**DOI:** 10.1371/journal.pmed.1000085

**Published:** 2009-05-19

**Authors:** Joseph T. Wu, Gabriel M. Leung, Marc Lipsitch, Ben S. Cooper, Steven Riley

**Affiliations:** 1 Department of Community Medicine and School of Public Health, Li Ka Shing Faculty of Medicine, The University of Hong Kong, Hong Kong SAR, China; 2 Department of Epidemiology and Department of Immunology and Infectious Diseases, Harvard School of Public Health, Boston, Massachusetts, United States of America; 3 Statistics, Modelling, and Bioinformatics Department, Centre for Infections, Health Protection Agency, London, United Kingdom; National Institutes of Health, United States of America

## Abstract

**Background:**

The effectiveness of single-drug antiviral interventions to reduce morbidity and mortality during the next influenza pandemic will be substantially weakened if transmissible strains emerge which are resistant to the stockpiled antiviral drugs. We developed a mathematical model to test the hypothesis that a small stockpile of a secondary antiviral drug could be used to mitigate the adverse consequences of the emergence of resistant strains.

**Methods and Findings:**

We used a multistrain stochastic transmission model of influenza to show that the spread of antiviral resistance can be significantly reduced by deploying a small stockpile (1% population coverage) of a secondary drug during the early phase of local epidemics. We considered two strategies for the use of the secondary stockpile: early combination chemotherapy (ECC; individuals are treated with both drugs in combination while both are available); and sequential multidrug chemotherapy (SMC; individuals are treated only with the secondary drug until it is exhausted, then treated with the primary drug). We investigated all potentially important regions of unknown parameter space and found that both ECC and SMC reduced the cumulative attack rate (AR) and the resistant attack rate (RAR) unless the probability of emergence of resistance to the primary drug *p_A_* was so low (less than 1 in 10,000) that resistance was unlikely to be a problem or so high (more than 1 in 20) that resistance emerged as soon as primary drug monotherapy began. For example, when the basic reproductive number was 1.8 and 40% of symptomatic individuals were treated with antivirals, AR and RAR were 67% and 38% under monotherapy if *p*_A_ = 0.01. If the probability of resistance emergence for the secondary drug was also 0.01, then SMC reduced AR and RAR to 57% and 2%. The effectiveness of ECC was similar if combination chemotherapy reduced the probabilities of resistance emergence by at least ten times. We extended our model using travel data between 105 large cities to investigate the robustness of these resistance-limiting strategies at a global scale. We found that as long as populations that were the main source of resistant strains employed these strategies (SMC or ECC), then those same strategies were also effective for populations far from the source even when some intermediate populations failed to control resistance. In essence, through the existence of many wild-type epidemics, the interconnectedness of the global network dampened the international spread of resistant strains.

**Conclusions:**

Our results indicate that the augmentation of existing stockpiles of a single anti-influenza drug with smaller stockpiles of a second drug could be an effective and inexpensive epidemiological hedge against antiviral resistance if either SMC or ECC were used. Choosing between these strategies will require additional empirical studies. Specifically, the choice will depend on the safety of combination therapy and the synergistic effect of one antiviral in suppressing the emergence of resistance to the other antiviral when both are taken in combination.

## Abstract 2

Mathematically simulating an influenza pandemic, Joseph Wu and colleagues predict that using a secondary antiviral drug early in local epidemics would reduce global emergence of resistance to the primary stockpiled drug.

## Editors' Summary

**Background:**

Every winter, millions of people catch influenza—a viral infection of the airways—and about half a million people die as a result. These seasonal “epidemics” occur because small but frequent changes in the viral proteins (antigens) to which the human immune system responds mean that an immune response produced one year provides only partial protection against influenza the next year. Influenza viruses also occasionally appear that contain major antigenic changes. Human populations have little or no immunity to such viruses so they can start deadly pandemics (global epidemics). The 1918–19 influenza pandemic, for example, killed 40–50 million people. The last influenza pandemic was in 1968 and many experts fear the next pandemic might strike soon. To prepare for such an eventuality, scientists are trying to develop vaccines that might work against an emerging pandemic influenza virus. In addition, many governments are stockpiling antiviral drugs for the large-scale treatment of influenza and for targeted prophylaxis (prevention). Antiviral drugs prevent the replication of the influenza virus, thereby shortening the length of time that an infected person is ill and protecting uninfected people against infection. Their widespread use should, therefore, slow the spread of pandemic influenza.

**Why Was This Study Done?:**

Although some countries are stockpiling more than one antiviral drug in preparation for an influenza pandemic, many countries are investing in large stockpiles of a single drug, oseltamivir (Tamiflu). But influenza viruses can become resistant to antiviral drugs and the widespread use of a single drug (the primary antiviral) is likely to increase the risk that a resistant strain will emerge. If this did happen, the ability of antiviral drugs to slow the spread of a pandemic would be greatly reduced. In this study, the researchers use a mathematical model of influenza transmission to investigate whether a small stockpile of a secondary antiviral drug could be used to prevent the adverse consequences of the emergence of antiviral-resistant pandemic influenza viruses.

**What Did the Researchers Do and Find?:**

The researchers used their model of influenza transmission to predict how two strategies for the use of a small stockpile of a secondary antiviral might affect the cumulative attack rate (AR; the final proportion of the population infected) and the resistant attack rate (RAR; the proportion of the population infected with an influenza virus strain resistant to the primary drug, a measure that may reflect the impact of antiviral resistance on death rates during a pandemic). In a large, closed population, the model predicted that both “early combination chemotherapy” (treatment with both drugs together while both are available) and “sequential multi-drug chemotherapy” (treatment with the secondary drug until it is exhausted, then treatment with the primary drug) would reduce the AR and the RAR compared with monotherapy unless the probability of emergence of resistance to the primary drug was very low (resistance rarely occurred) or very high (resistance emerged as soon as the primary drug was used). The researchers then introduced international travel data into their model to investigate whether these two strategies could limit the development of antiviral resistance at a global scale. This analysis predicted that, provided the population that was the main source of resistant strains used one of the strategies, both strategies in distant, subsequently affected populations would be able to reduce the AR and RAR even if some intermediate populations failed to control resistance.

**What Do These Findings Mean?:**

As with all mathematical models, the accuracy of these predictions depends on the assumptions used to build the model and the data fed into it. Nevertheless, these findings suggest that both of the proposed strategies for the use of small stockpiles of secondary antiviral drugs should limit the spread of drug-resistant influenza virus more effectively than monotherapy with the primary antiviral drug. Thus, small stockpiles of secondary antivirals could provide a hedge against the development of antiviral resistance during the early phases of an influenza pandemic and are predicted to be a worthwhile public-health investment. However, note the researchers, experimental studies—including determinations of which drugs are safe to use together, and how effectively a given combination prevents resistance compared with each drug used alone—are now needed to decide which of the strategies to recommend in real-life situations. In the context of the 2009 global spread of swine flu, these findings suggest that public health officials might consider zanamivir (Relenza) as the secondary antiviral drug for resistance-limiting strategies in countries that have stockpiled oseltamivir.

**Additional Information:**

Please access these Web sites via the online version of this summary at http://dx.doi.org/10.1371/journal.pmed.1000085.

- The US Centers for Disease Control and Prevention provides information about influenza for patients and professionals, including specific information on pandemic influenza and on influenza antiviral drugs
- The World Health Organization provides information on influenza (in several languages) and has detailed guidelines on the use of vaccines and antivirals during influenza pandemics
- The UK Health Protection Agency provides information on pandemic influenza
- MedlinePlus provides a list of links to other information about influenza (in English and Spanish)

## Introduction

Large-scale antiviral treatment and targeted prophylaxis may provide substantial public health benefits by slowing the spread of pandemic influenza [1]–[7]. Although it is not certain that existing antivirals will be effective against the eventual pandemic strain, many countries are investing in large stockpiles of a single drug (oseltamivir [Tamiflu]) [8]. Such massive use of a single antiviral will substantially increase the risk of emergence of resistant strains. For H3N2 strains, de novo emergence of resistance occurs in 0.4% of outpatient adults and 5.5% of outpatient children treated with oseltamivir [9]. If the rate of de novo emergence is similar for the pandemic strain, resistance will certainly emerge under large-scale antiviral intervention [5]. Although most oseltamivir-resistant H3N2 strains are less fit (i.e. less transmissible) than the wild type [10] and therefore cannot spread widely [11], there is no guarantee that this will be the case for the yet-to-be-observed pandemic strain. Indeed, the recent establishment of oseltamivir-resistant H1N1 viruses suggests that oseltamivir-resistant strains do not necessarily incur fitness costs [12]–[18]. The spread of antiviral resistance during a pandemic will substantially reduce the effectiveness of antiviral intervention [5],[19]–[21]. Despite this potential threat, countries stockpiling antivirals have not yet declared any strategies to hedge against the risk of antiviral resistance. Here, we test the hypothesis that a small stockpile of a secondary antiviral drug could be used to effectively mitigate the adverse consequences of the emergence of resistant influenza strains.

## Methods

### Natural History and Transmissibility

We adopted the natural history model (Figure A in Text S1) used in our previous study of influenza pandemic mitigation [7]. Infected individuals progressed from S (susceptible) through E (latent) to P (infectious and presymptomatic) to I (infectious and symptomatic, with probability *p_s_* = 0.67) or A (infectious and asymptomatic, with probability 1−*p_s_* = 0.33) and finally to R (removed). Each symptomatic individual was treated with antivirals with probability *p_T_* at the onset of symptoms. In the base case, we assumed that the basic reproductive number was *R*_0_ = 1.8 [2],[3],[22], the generation time was *T_G_* = 2.6 d [3], the proportion of infections in which the infector was not symptomatic was *θ* = 0.3 [7], and the treatment probability was *p_T_* = 0.4. Under antiviral intervention, the effective reproductive number of the wild type was
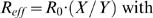


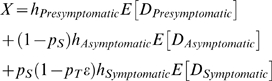


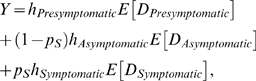
where *h_i_* was the relative infectiousness of disease stage *i*, *E*[*D_i_*] was its mean duration, and *ε* = 66% was the efficacy of antivirals in reducing infectiousness [23],[24]. In the absence of resistance, antiviral treatment eliminated *p_T_ε*(1−*θ*) = 18.5% of transmission per infected individual on average. This level of large-scale antiviral intervention was not sufficient to eradicate the epidemic in its early stages, but could nonetheless be useful in reducing the total number of persons infected as the epidemic sweeps through the population, while still allowing enough people to become infected so that herd immunity would prevent a second epidemic [25].

### Multiple Drugs and Strains

Our objective was to investigate the possible benefits of multidrug strategies over monotherapy in terms of reducing the impact of treatment-induced resistance. Therefore, we considered antiviral therapies in a generic sense: drug A was the primary antiviral in national stockpiles and drug B was the secondary antiviral to be used to reduce the emergence and spread of resistance. In the context of currently available influenza antivirals and pandemic preparedness, drug A would be oseltamivir because that is the drug that has been stockpiled. Drug B could be zanamivir (Relenza) or an adamantane derivative (amantadine [Symmetrel] or rimantadine [Flumadine]). Although there are few data from humans (or from good animals models of human influenza) that combination antiviral therapy can reduce the emergence of resistance [26], a recent study showed that combination chemotherapy with oseltamivir and amantadine substantially reduced the emergence of drug-resistant influenza variants in vitro [27]. Further, at the population level, the transmission of strains that are resistant to only one of the drugs will likely be hindered by combination chemotherapy [28],[29]. As a key premise of this study, we assumed that the wild-type pandemic strain was sensitive to both drugs with probabilities of emergence of resistance *p_A_* and *p_B_*. If a wild-type case was treated with combination chemotherapy, the probabilities of emergence of resistance were reduced by a synergy proportion *s* (Figure B in Text S1). The degree of synergy would likely depend on the specific classes of drugs that are used in combination, e.g., oseltamivir and zanamivir versus oseltamivir and an adamantane derivative (see Discussion). If the strain under treatment was already resistant to one drug, we assumed that combination chemotherapy had no effect in reducing emergence rates. Combination chemotherapy reduced infectiousness by *ε* = 66% (i.e., same as monotherapy) unless the treated case was resistant to both drugs. Also, resistant strains had the same natural history as the wild type and no fitness cost (all strains were equally transmissible). In our sensitivity analysis, we considered the impact of fitness cost on the spread of antiviral resistance and the effectiveness of our hedging strategies (see Discussion). Recovery from infection with any strain provided immunity to all strains.

### Global Transmission Network

We used a discrete-time stochastic multistrain transmission model to simulate the spread of pandemic influenza in a global network of 105 major cities (see Text S1). We assumed homogeneous mixing within each city. The simulation proceeded with a time-step of 0.25 d, which was small enough to be accurate (an independent discrete-event simulation was used to calibrate the time-step; see Text S1). The network was parameterized with the travel and city data previously used to study the international spread of pandemic influenza [30]. We assumed that the average duration of travel was 7 d and that individuals on travel did not receive antiviral treatment. See Text S1 for algorithmic details of the simulations.

### Outcomes

Our main outcome variables were attack rate (AR, the final proportion of the population infected, both with and without symptoms) and resistant attack rate (RAR, the final proportion infected with a strain resistant to the primary antiviral). AR and RAR are both important measures for the impact of the spread of antiviral resistance. For example, AR reflects the overall societal impact (e.g., burden on the health care system, work absenteeism) posed by the pandemic; RAR indicates the number of infections not treatable by the primary antiviral and therefore may reflect the impact of antiviral resistance on pandemic mortality.

## Results

### Monotherapy in a Closed Population

We investigated the dynamics of resistance emergence and mitigation in the first large population to implement large-scale antiviral treatment (assumed to have 6.8 million individuals, the size of Hong Kong). Our baseline scenario was that resistance to the primary antiviral (drug A) emerged with probability *p_A_* = 0.01 per treated symptomatic individual. Typically, for *R*_0_ = 1.8, the demand created by a treatment probability of *p_T_* = 0.4 was satisfied by a stockpile sufficient to treat 20% of the population (i.e., stockpile coverage of 20%).

**Figure 1:**
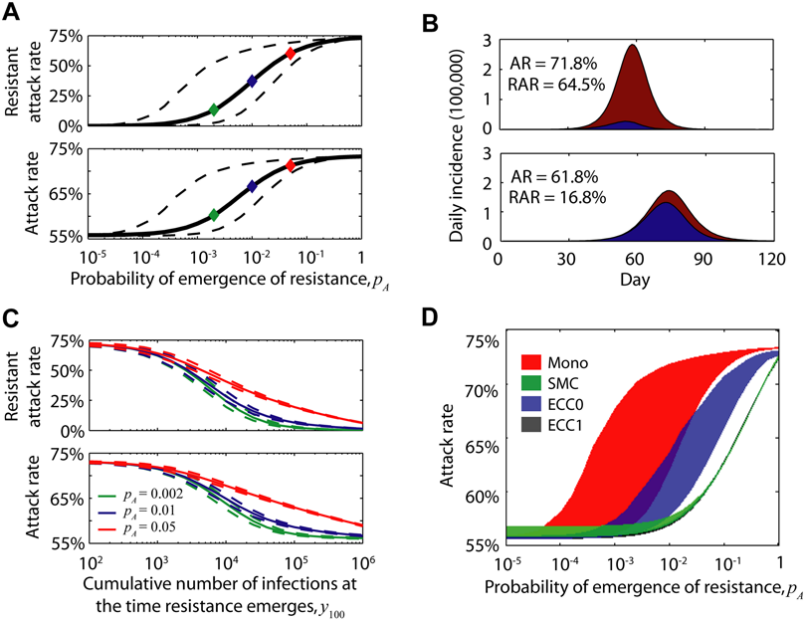
Dynamics of resistance emergence and mitigation in a single population. Outcome variables were calculated using 10,000 simulations of 365 d in a population of 6.8 million individuals. See main text for baseline natural history parameters. (A) The baseline monotherapy scenario was associated with substantial stochasticity for a wide range of values of *p_A_* (dashed lines, 95% prediction intervals; colored diamonds indicate scenarios for emergence rates used in [C]). (B) Two stochastic realizations of the single-population epidemic with *p_A_* = 0.01 (brown shaded area corresponds to resistance incidence; blue shaded area corresponds to wild-type incidence; upper graph, resistance emerged early; lower graph, resistance did not emerge early). (C) AR and RAR as functions of the cumulative number of wild-type infections at the time resistance emerged (dashed lines, 95% prediction intervals; colors correspond to the value of *p_A_* as per diamonds in [A]; in the absence of interventions AR = 73% and RAR = 0%; in the absence of resistance, AR = 56% and RAR = 0%). (D) Efficacy of ECC (antiviral synergies of *s* = 0 and *s* = 1) and SMC in reducing the attack rate (shaded areas, 95% prediction intervals; purple shading indicates overlap between red and blue shades). Note that the results for ECC1 correspond to the thin gray area that forms the lower border of the green area.

Under monotherapy, there was substantial stochastic variation in AR and RAR among different stochastic realizations, even in a large population, across a wide range of probabilities of emergence of resistance *p_A_* (Figure 1A). This variation existed because AR and RAR were sensitive to the time at which resistance first emerged and started to spread: if resistance emerged early in the epidemic, the resistant strain dominated transmission (Figure 1B, upper graph). Conversely, the spread of resistance was limited if resistance did not emerge early (Figure 1B, lower graph). Because the number of treated cases was small during the early stages when the epidemic size was still small, the time at which resistance first emerged had substantial stochastic variation, hence the stochastic variation in AR and RAR seen in Figure 1A.

In view of the dramatic impact of early emergence of resistance on AR and RAR, we considered next the effect of a deterministic delay in the emergence of resistance (Figure 1C). We defined *t*_100_ to be the time at which the number of resistant cases first reached 100 and *y*_100_ to be the cumulative number of wild-type infections at *t*_100_. As *y*_100_ increased, the final attack rates AR and RAR decreased because (i) there were more wild-type infectors competing with resistant infectors for susceptible individuals, and (ii) the number of susceptible individuals available for the resistant strain decreased. While the reduction in AR might appear to be limited (a maximal drop of 17% from 73% when all cases were resistant to 56% when resistance was absent; Figure 1C, lower graph), the corresponding reduction in RAR could be very large (Figure 1C, upper graph). A large drop in RAR necessarily implies that more infected cases will be sensitive to antiviral treatment, a factor which is likely to be critical for minimizing case fatalities.

The results in Figure 1C suggested that unless the probability of resistance emergence was very high, the spread of resistance could be substantially reduced if emergence could be delayed until the cumulative number of wild-type infections had reached 10^5^ (1.5% of the population). This threshold was robust against variations in population size (Figure J in Text S1). In principle, such a delay could be achieved simply by postponing the launch of antiviral intervention (with only a minimal cost in terms of additional infections). However, because of the high therapeutic value of antivirals, even a short deliberate delay is undesirable and likely unethical. Therefore, as an alternative, we propose the deployment of a small stockpile of a secondary antiviral during the early phase of the local epidemic.

### Small Stockpiles of a Secondary Antiviral

We considered two alternative strategies for the deployment of the secondary antiviral. Under early combination chemotherapy (ECC), at the start of the epidemic, symptomatic individuals were treated (with probability *p_T_*) with both primary antiviral (drug A, large stockpile) and secondary antiviral (drug B, small stockpile) in combination until the stockpile of drug B was depleted. After that time, treatment comprised only drug A. Individuals infected with the wild-type strain and treated with both drugs could generate strains resistant to either drug (Figure B in Text S1). However, probabilities of resistance emergence were reduced by a synergy factor *s* [27],[31],[32]. Under sequential multidrug chemotherapy (SMC), individuals received only drug B until the secondary stockpile was exhausted, after which time treatment comprised only drug A. We considered a stockpile of drug B sufficient to treat 1% of the population (i.e. stockpile coverage of 1%). This was small compared with the coverage of drug A required for large-scale antiviral intervention (∼20%), but was sufficient to implement SMC or ECC.

Both SMC and ECC were effective in reducing the spread of resistance across a wide range of *p_A_* when the probability of resistance emergence to the secondary antiviral was *p_B_* = 0.01 (Figure 1D; Table 1). Given the absence of zanamivir resistance in naturally occurring influenza strains, *p_B_* = 0.01 may be viewed as a reasonable upper bound for zanamivir, and our baseline scenario may be interpreted as zanamivir being the secondary antiviral. When synergy was perfect (*s* = 1), ECC was the most effective, reducing the mean AR from 67% to 57% (RAR from 38% to 3%) (see Table 1). Even when synergy was absent (*s* = 0), ECC reduced the mean AR from 67% to 59% (RAR from 38% to 9%): combination chemotherapy reduced the infectiousness of cases that were resistant to drug A but sensitive to drug B, thereby decreasing the competitiveness of these A-resistant cases against the wild types unless these cases became dually resistant. However, when synergy was low, the effectiveness of ECC deteriorated as *p_B_* increased (Table 1; Table B and Figure C in Text S1). For example, when *s* = 0, *p_A_* = 0.01, and *p_B_* = 0.05, ECC reduced the mean AR from 67% to only 61% (RAR from 38% to 14%). In contrast, SMC remained effective even when *p_B_* was high because it delayed the emergence of A-resistant cases by simply substituting A-monotherapy with B-monotherapy during the early stage (Table 1; Table B and Figure C in Text S1). In summary, ECC was superior to SMC when synergy was high (enhancing the specific benefit of combination therapy in reducing emergence) or when *p_A_* was low (in which case the performance gap was small). Otherwise, SMC was more successful, because it reliably delayed the emergence of drug-A resistance by not using drug A at the start of antiviral intervention.

**Table 1:** Example ARs and RARs under SMC and ECC with synergy of 0 (ECC0) or 1 (ECC1).

*p_A_*	AR or RAR	Mono	SMC	ECC1	ECC0 with *p_B_* = 0.01	ECC0 with *p_B_* = 0.05	ECC0 with *p_B_* = 0.3
0.001	AR	58 (56, 68)	57 (56, 57)	56 (56, 56)	56 (56, 57)	56 (56, 60)	58 (56, 66)
	RAR	7 (1, 42)	0 (0, 0)	0 (0, 0)	1 (0, 4)	2 (0, 11)	5 (1, 33)
0.01	AR	67 (62, 72)	58 (57, 58)	57 (57, 57)	59 (58, 64)	61 (58, 68)	65 (60, 71)
	RAR	38 (18, 64)	2 (2, 3)	3 (3, 3)	9 (5, 24)	14 (6, 43)	29 (12, 59)
0.1	AR	72 (71, 73)	63 (63, 63)	63 (63, 63)	67 (66, 71)	69 (67, 72)	72 (70, 73)
	RAR	66 (60, 71)	17 (15, 18)	18 (18, 18)	39 (32, 57)	49 (38, 65)	62 (54, 69)

Note: ARs and RARs are shown as means followed by the 95% prediction intervals. Three probabilities of resistance emergence for drug B are shown here: *p_B_* = 0.01, 0.05, and 0.3. Under SMC and ECC1 (ECC with perfect synergy), AR and RAR are insensitive to the value of *p_B_* in this range (see Table S2), hence only one set of outcomes is shown.

**Figure 2:**
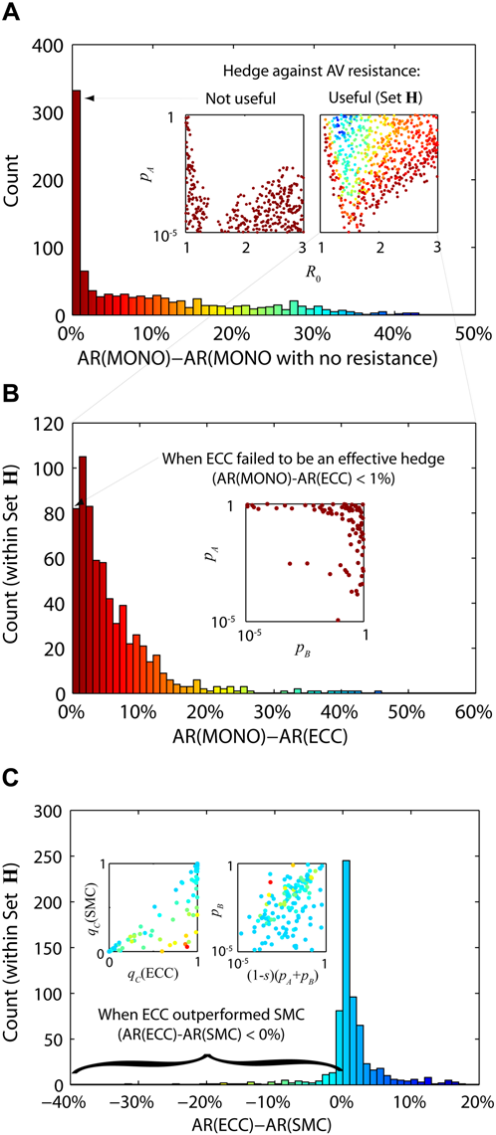
Sensitivity analysis. We used Latin-hypercube sampling to generate 1,000 combinations of the following parameters: basic reproductive number *R*_0_, linear scale on interval (1,3); generation time *T_G_*, linear, (2,4) d; proportion of infections in which the infector was not symptomatic *θ*, linear, (0,0.3); proportion of *θ* in which the infector was never symptomatic, linear, (0,1); probability of emergence of resistance to the primary antiviral *p_A_*, log, (10^−5^, 1); probability of emergence of resistance to the secondary antiviral *p_B_*, log, (10^−5^, 1); synergistic effects of combination therapy in reducing the rates of emergence of resistance *s*, linear, (0,1) (see the left column of Figure E in Text S1 for 1−*s* on log scale, (10^−7^, 1)). A probability of treatment of *p_T_* = 0.4 was used throughout (see the right column of Figure E in Text S1 for *p_T_* = 1). For each parameter combination, we estimated the mean attack rate (from 2,500 realizations) for: monotherapy with resistance, AR(MONO); monotherapy without resistance, AR(MONO/R), i.e., *p_A_* = *p_B_* = 0; early combination chemotherapy, AR(ECC); and sequential multidrug chemotherapy, AR(SMC). (A) *p_A_* and *R*_0_ determined the usefulness of a hedge against the emergence of resistance. Main chart, frequency of parameter combinations versus the increase in monotherapy AR due to resistance, AR(MONO)−AR(MONO/R); inset charts, parameter subsets for AR(MONO)−AR(MONO/R)<1% (left) and >1% (right, Set H), points are colored as per the *x*-axis values in main chart. (B) If a hedge was useful (i.e., for those parameter combinations in Set H), ECC failed if *p_A_* or *p_B_* or both were large and synergy was not high. Main chart, frequency of parameter combinations versus the marginal benefit of ECC over monotherapy, AR(MONO)−AR(ECC); inset, distribution of parameter combinations in the *p_B_*-*p_A_* plane for which AR(MONO)−AR(ECC)<1%. Note: The colors here are not related to those in (A) (C) SMC performed better than ECC except when combination therapy results in very low probability of resistance emergence (e.g., very high synergy), yet drug B monotherapy has a high risk of emergence, rendering drug B monotherapy unsuitable and combination therapy highly effective. The proportion of scenarios for which ECC outperformed SMC (i.e. AR(ECC)−AR(SMC)<0%) was 22%. In a subset of such scenarios, ECC, but not SMC, had a high probability of achieving containment. Main chart, frequency of parameter combinations versus AR(ECC)−AR(SMC). Containment here was defined as an attack rate of <3%. Inset charts, distribution of parameter combinations for which AR(ECC)−AR(SMC)<0%. Inset left, *q_c_*(SMC) and *q_c_*(ECC) were the proportion of realizations with attack rate <3% under SMC and ECC; inset right, *q_c_*(SMC)≪*q_c_*(ECC) when *p_B_* was high and (1−*s*)(*p_A_*+*p_B_*) (the probability of resistance emergence in a wild-type case under combination chemotherapy) was low.

We conducted an extensive sensitivity analysis to investigate explicitly how the benefits of ECC and SMC in hedging against resistance varied across the space of unknown parameter values (Figure 2). The potential value of a hedge against emergence of resistance was assessed by comparing the attack rate under monotherapy with the attack rate under monotherapy if resistance were absent. This value was greatest at intermediate values of *R*_0_, where large-scale antiviral intervention was able to make a significant reduction in attack rate (up to 40%) yet the spread of resistance was likely under monotherapy. In all cases, a hedge was more useful for higher values of *p_A_* (Figure 2A). For values of *R*_0_ and *p_A_* for which a hedge was useful, ECC was an effective hedge unless values of either *p_A_* or *p_B_* were very high, or both were moderately high (Figure 2B). Finally, for most values of *R*_0_ and *p_A_* for which a hedge was useful, SMC performed better than ECC unless containment (here defined as AR<3%) was likely under ECC but not SMC, which was the case when *p_B_* was high but the probability of resistance emergence in a wild-type case under combination chemotherapy, (1−*s*)(*p_A_*+*p_B_*), was low, e.g., with very high synergy (Figure 2C).

The choice between ECC and SMC is particularly sensitive to the synergy parameter *s*. In the sensitivity analysis presented above, we assumed treatment coverage of *p_T_* = 0.4 and considered a uniform distribution for possible values of the synergy parameter *s* between 0 and 1. This choice favored SMC over ECC in approximately eight out of every ten scenarios we considered (Figure 2). Some experts believe that synergy is likely to be in the range of 0.95 or greater (i.e., combination chemotherapy reduces the probabilities of emergence of single-drug resistance by more than 20 times). We repeated the sensitivity analysis (see Figures E and F in Text S1) with higher values of synergy and with the extreme assumption of perfect treatment coverage (*p_T_* = 1). Under these assumptions ECC was more effective than SMC in approximately six out of every ten scenarios considered (see Discussion).

### Global Spread of Disease and Resistance

**Figure 3:**
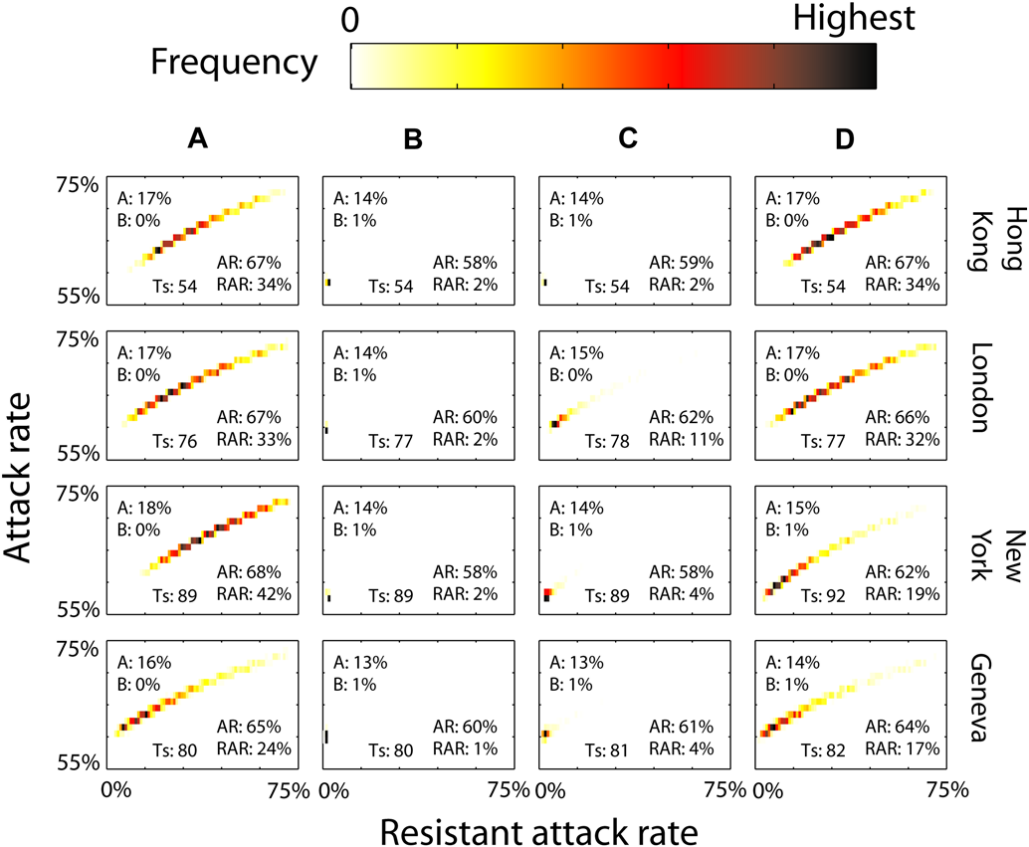
SMC in a global network of 105 cities. Hong Kong (HK) is the source of infection in the network with 30 wild-type seeds on day 0. Twenty-eight cities implement large-scale antiviral intervention: Hong Kong, London, New York, Geneva, and 24 other cities (randomly chosen for each stochastic realization). Cities that implemented SMC had a drug B stockpile coverage of 1%. In this 4-by-4 chart panel, each row corresponds to a city (Hong Kong, London, New York or Geneva) and each column (A–D) corresponds to a different scenario. Each panel is the 2-D histogram (1,000 realizations) of attack rate (*y*-axes, 55%–75%) and resistant attack rate (*x*-axes, 0%–75%) for a given city and scenario. A bin size of 1% is used on both axes. The color for each bin indicates the frequency for that bin within that chart (see legend), i.e., same color in different panels does not indicate same frequency. The mean AR and RAR are shown at the bottom-right of each chart. The number *T_s_* at the bottom of each chart is a measure of the time at which the epidemic had clearly taken off in each city: the mean time at which 1% of the population were infected. The numbers *A* and *B* at the upper-left indicate the mean amount of drugs A and B used (in terms of stockpile coverage, which means the number of treatment courses per capita). Four scenarios are shown: (A) HK and all 27 cities implemented monotherapy. (B) HK and all 27 cities implemented SMC. (C) HK, New York. Geneva and 11 other randomly chosen cities implemented SMC; London and 13 other randomly chosen cities implemented monotherapy. (D) Same as (C) except that HK did not implement SMC.

In the global context, the analysis described so far applies to a “source population,” one whose epidemic takes off early enough in the pandemic so that the importation of resistant viruses is not a significant risk. However, given the high connectivity among populations in the global network, it was not apparent that ECC or SMC would also be effective for other populations (downstream populations), because they would more likely be seeded by resistant strains than would source populations. Therefore, to investigate the effectiveness of ECC and SMC at a global scale, we simulated the international spread of a multistrain influenza pandemic over a network of 105 major cities with Hong Kong as the source [30]. We assumed the same baseline natural history and emergence parameters as above and we selected 28 out of the 105 populations to have antiviral stockpiles (Figure 3). Conclusions drawn from this scenario are robust against the number of populations implementing antiviral intervention and the choice of source population (see Text S1 and Figure G therein). Results are presented only for the comparison between SMC and monotherapy. However, results comparing ECC to monotherapy are not qualitatively different.

If only monotherapy was used, the importation of resistance promoted the spread of the resistant strain and downstream populations had higher ARs and RARs, e.g., New York had a higher AR and RAR than London because the pandemic reached New York later, with a higher proportion of introduced infections being resistant (Figure 3A). Population size also played a role. The small population of Geneva had a smaller RAR than London even though the two were hit at approximately the same time: smaller populations were less vulnerable to the local emergence of antiviral resistance because fewer cases were treated with drug A. We note that our city population sizes are only proxy measures for entire local populations which feed into major airports (see Discussion).

If all 28 populations that had stockpiles of antivirals implemented SMC rather than monotherapy, reductions in AR and RAR in these populations were similar to those in a single source population (Figure 3B). Therefore, the connectedness of cities had little impact on the effectiveness of SMC if all populations that implemented large-scale antiviral interventions adopted SMC. The effectiveness of SMC was attenuated (but was still significant) if only half of these 28 selected populations adopted SMC (Figure 3C). Interestingly, in this scenario, those populations that implemented only monotherapy (e.g., London) still benefited from the implementation of SMC in the other populations because fewer resistant cases were circulating within the network.

The source population was the key to the robustness of SMC as a resistance-limiting strategy at the global scale. If the source population implemented only monotherapy, then SMC had little benefit in any downstream population (Figure 3D). If the source population did implement SMC, then downstream populations benefited (through lower AR and RAR) under a surprisingly broad range of conditions (Figure G and H in Text S1). In particular, if most of the immediate neighbors of the source population were able to control resistance, then other cities (not directly connected to the source) benefited because they were seeded predominantly by wild-type virus. Only if most of the neighbors of the source population failed to control resistance did the benefit of SMC at the source fail for other downstream populations. This in turn occurred only if most neighbors implemented monotherapy and *p_A_* was large (see Text S1).

## Discussion

Our model predicts that the spread of treatment-induced antiviral resistance during an influenza pandemic can be effectively reduced by deploying a small stockpile of a secondary drug during the early phase of local epidemics. By investigating all potentially important regions of unknown parameter space we found that both ECC and SMC reduced the cumulative AR and the RAR unless the probability of emergence of resistance to the primary drug was so low that resistance was unlikely to be a problem or so high that resistance emerged as soon as primary drug monotherapy began. Using a global model of large cities, we found that as long as populations that were the main source of resistant strains employed these strategies (SMC or ECC), then those same strategies were also effective for populations far from the source even when some intermediate populations failed to control resistance.

The implications of our results are straightforward: a small stockpile of a secondary antiviral could be used to hedge against the threat of drug-induced antiviral resistance during the next influenza pandemic in terms of reducing the overall AR and the RAR (and hence significantly protect the therapeutic value of the primary antiviral). We have proposed two ways to use such an antiviral, either as combination therapy (ECC) or as sequential monotherapy (SMC). Crucially, under a wide range of possible parameter values, both are superior to the current policy of monotherapy. Therefore, we recommend that a stockpile of a second drug be assembled for use in likely source populations at least (we discuss choices for this drug below), and downstream populations where possible, and that studies be commenced to assess the key drivers of the choice between SMC and ECC. Those drivers are the safety of combination regimens and the degree of synergy between the drugs in vivo. Safety can and should be assessed prior to a pandemic, while synergy could be studied further using seasonal and zoonotic strains of influenza A to provide a basis for studies of a pandemic strain when it emerges.

At the global scale, the success of these strategies requires source populations to minimize their prevalence of resistance so that downstream populations will not be seeded by large numbers of resistant cases. This prediction suggests that likely downstream populations have a strong incentive to assist likely source populations, perhaps with the WHO managing a globally vested stockpile of secondary antivirals. Although it seems that SMC and ECC reduce AR by only a small margin (e.g., by about 10% in Figure 3), their major predicted benefit is the substantial reduction in RAR (e.g., to as low as RAR = 0% in Figure 3) and hence in mortality related to antiviral resistance.

More specifically, in the context of currently implemented pandemic preparedness plans, oseltamivir is the only primary antiviral. Adamantane derivatives and zanamivir are possible secondary antivirals for the hedging strategies we propose here (SMC and ECC). Despite high rates of emergence of resistance to amantadine (e.g., 30% in outpatient adults [26]), there is in vitro evidence of significant synergy between it and oseltamivir [27],[28]. Also, because the two drugs have independent biological mechanisms, they are likely to have independent resistance profiles. Therefore, ECC could be implemented with an adamantane derivative as the secondary antiviral to a primary stockpile of oseltamivir. Our results imply that even when synergy was only moderate, ECC would be very effective in hedging against oseltamivir resistance despite the high rates of resistance emergence for adamantane derivatives (Figure 2; Figure C in Text S1). However, because side effects due to adamantane derivatives are non-negligible, side effects from a combination of an adamantane derivative and oseltamivir could lower the levels of compliance and must be carefully evaluated before implementation.

Zanamivir is a neuraminidase inhibitor that is comparable to oseltamivir in efficacy against seasonal influenza [23],[33],[34]. Circulating oseltamivir-resistant strains are sensitive to zanamivir [35],[36]. Although cross-resistance is a theoretical concern, zanamivir and oseltamivir bind differently at the neuraminidase catalytic site and exhibit different drug resistance profiles [17],[37],[38]. Although zanamivir is not licensed for treatment in children younger than 7 years of age and may not be able to reduce infectiousness, these drawbacks have little impact on the effectiveness of SMC and ECC (see Text S1). Therefore, zanamivir should be an effective secondary antiviral for both SMC and ECC.

Nonetheless, there are some potential implementation issues. Despite the comparable efficacies of oseltamivir and zanamivir [33],[34], zanamivir's potentially more cumbersome delivery method (powder inhalation) and higher cost have caused current stockpiles to emphasize oseltamivir. For SMC with zanamivir as the secondary antiviral, a potential hurdle is that the current de facto second-choice therapy would need to be deployed first, and this may lower the level of compliance. For ECC with zanamivir as the secondary antiviral, a safe and effective combination protocol for zanamivir and oseltamivir would need to be developed. As additional antivirals are developed in the future, key features determining an agent's suitability for use in resistance hedging strategies will be its effectiveness for monotherapy (to permit SMC), and its safety in combination with oseltamivir and synergy in preventing resistance (to permit ECC).

We have used a stochastic model throughout this study. In Text S1, we formulated a deterministic version of our stochastic model and compared results for the key scenarios considered in Figure 1 and Table 1 (see also Figure N and Table C in Text S1). Results were qualitatively similar but the deterministic model did not accurately estimate the mean behavior of the stochastic model (in terms of AR and RAR). Thus, while a deterministic model would have sufficed to show that delaying the use of antivirals could reduce AR and RAR (compare Figure 1 with Figure N in Text S1), a stochastic model is more appropriate as it allows us to quantify the role of chance in the emergence and spread of antiviral resistance (Figure 1A and 1C) and international spread of pandemic influenza (where chance effects dominate in the early stages of the epidemic in each city and in the seeding of each city's epidemic [1],[30]).

Our study has several limitations due to the assumptions we used. First, we assumed that the pandemic virus was sensitive to both drugs when it arrived at the first major city of the global air-travel network. Second, we assumed that resistance was induced by treatment only and ignored other ways by which resistance might emerge (e.g., reassortment with circulating seasonal strains). Third, we assumed that resistant strains had no fitness cost and might therefore overestimate the threat of antiviral resistance. Fourth, the 105-city air travel network that we used might not accurately reflect the global spread of pandemic influenza. Finally, there was not sufficient evidence to choose definitively between the two alternative strategies, SMC and ECC. We discuss the implications of each of these limitations below.

A key premise of this study is that the wild-type pandemic strain was sensitive to both drugs. The effectiveness of our hedging strategies will be much reduced if the pandemic strain acquires resistance to the primary antiviral early at the source by means that are independent of drug pressure, e.g., de novo resistance among pandemic viruses or reassortment with a circulating seasonal strain that is resistant. As shown in the global model (Figure 3), even a small number of resistant pandemic cases at the early phase of local epidemics would greatly increase the spread of resistance and diminish the effectiveness of SMC and ECC (see Figure M in Text S1 for further illustrations). We did not explicitly consider the cocirculation of an existing human influenza strain which was resistant to one or more of the antivirals (currently circulating H1N1 with the H274Y mutation [39]), which would increase the probability of the emergence of resistant pandemic strains via reassortment in a human host. We have conducted a supplementary analysis to show that the effect of this dynamic interaction with a seasonal strain would likely be small compared to the level of drug-induced resistance considered in this study (see the section “Resistance emergence due to reassortment” in Text S1 and Figure K therein). Therefore, the recent establishment of the oseltamivir-resistant H1N1 strain [12]–[18] does not affect our conclusions.

We used a four-strain model in which all strains were fully fit. The strain infecting a given infectious individual could be sensitive to (i) both drugs, (ii) only drug A, (iii) only drug B, or (iv) neither drug. Because the objective of this study was to devise strategies for hedging against the risk of antiviral resistance, we assumed fully fit resistant strains as the worst-case scenario and showed that SMC and ECC with a small stockpile of secondary antivirals were effective even under such extreme scenarios. We conducted an additional analysis regarding the implications of less fit emergent strains and found that the spread of antiviral resistance under monotherapy was limited if resistant strains were at most 80% as transmissible as the wild type (see Figure L in Text S1). We chose not to represent individual mutations of the influenza virus despite the fact that a single mutation (or a small set of mutations) might confer resistance to multiple drugs and that the evolutionary pathway to more fit mutant strains may be via less fit mutant strains (i.e., stepwise evolution). Implicitly, we assumed that the time scales of successful mutational pathways would be short compared to the key timescales of our study: transient, less fit strains leading to important fit strains would become extinct in times far shorter than the duration of a single local epidemic. While we appreciate that a more complex model structure might be useful to investigate other interesting hypotheses, it seems unlikely that more than four strains could persist for substantial periods of time and be epidemiologically significant.

We used our model of 105 large, well-mixed cities connected by a flight network as a proxy for the global spread of infection, which is consistent with a previous analysis of the seasonal spread of influenza [40] and other theoretical studies of pandemic influenza [3],[4], i.e., each city represents the entire local population which would normally use an airport of that city. Therefore, our results with regard to the population size of each city must be treated with some caution because the quoted size of the city may not correspond directly to the size of the population that would enter the flight network at that point.

**Figure 4:**
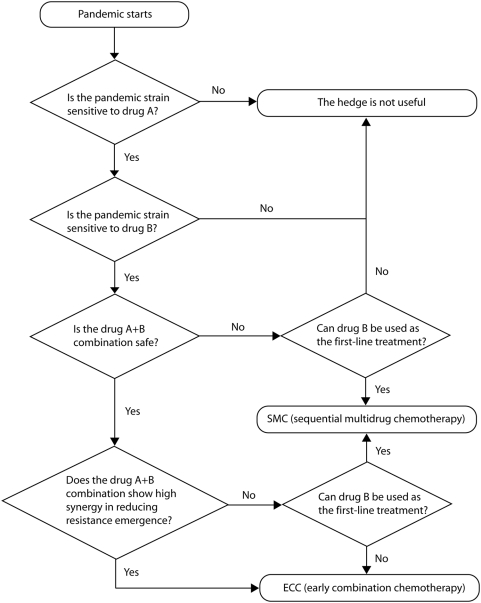
A decision flow chart for determining the optimal use of a secondary antiviral for hedging against the threat of antiviral resistance against the primary drug during an influenza pandemic. Some of the data needed can be collected before the pandemic strikes, e.g., whether the side effects of combination chemotherapy are tolerable. Other data needed can be collected in real time after the pandemic virus has been observed, e.g., drug sensitivity of the pandemic virus and whether combination chemotherapy shows high synergy in reducing emergence of resistance for the pandemic strain.

We present a flowchart (Figure 4) that could be used to choose between SMC and ECC. If initial reports of the transmissibility of the novel virus suggest that containment is unlikely to be achieved with antiviral intervention, SMC is the safer of the two strategies because (i) it does not depend on the synergistic effects of the two drugs, (ii) it does not depend on a low probability of resistance emergence to the secondary antiviral, and (iii) it does not require individuals to take two drugs in combination. However, if containment with antivirals appears to be feasible, and it can be demonstrated that the primary and secondary antiviral can be taken safely and effectively in combination, then ECC would be the safer strategy because it maximizes the probability of containment.

We chose to investigate incremental additions to current pandemic preparedness plans, rather than more extreme strategies. If safe combinations of antivirals were developed, then the use of combination chemotherapy for the full duration of national epidemics would necessarily be more effective than ECC. However, the additional investment in drug B beyond the levels we considered here is unlikely to be justified by the relatively small marginal benefit (Figure I in Text S1). Further, we have identified plausible parameter combinations for which full combination chemotherapy would perform less well than SMC.

For nations that are currently maintaining a stockpile of a single antiviral with the intention of mitigating the effects of the next influenza pandemic, the inclusion of a small stockpile of a second antiviral does not represent a substantial additional investment. However, our model predicts substantial potential benefits for populations that use a second antiviral early in their epidemic to limit the impact of the emergence of resistance. These benefits are unlikely to be attenuated by the global spread of resistance unless early users of antiviral intervention fail to control resistance. Hence, a small stockpile of a secondary antiviral therapy is an attractive public health hedge.

## Supporting Information

Text S1 **Algorithms and additional sensitivity analyses.**
(3.66 MB PDF)

Video S1 **Four stochastic realizations of the global spread of pandemic influenza and antiviral resistance for the scenario in** **Figure 3A** **.** For graphical clarity, Hawaii and Wellington are not shown. The city markers would overlap significantly if the entire world map were shown.
(4.44 MB MOV)

Video S2 **Four stochastic realizations of the global spread of pandemic influenza and antiviral resistance for the scenario in** **Figure 3B** **.** For graphical clarity, Hawaii and Wellington are not shown. The city markers would overlap significantly if the entire world map were shown.
(4.30 MB MOV)

Video S3 **Four stochastic realizations of the global spread of pandemic influenza and antiviral resistance for the scenario in** **Figure 3C** **.** For graphical clarity, Hawaii and Wellington are not shown. The city markers would overlap significantly if the entire world map were shown.
(4.32 MB MOV)

Video S4 **Four stochastic realizations of the global spread of pandemic influenza and antiviral resistance for the scenario in** **Figure 3D** **.** For graphical clarity, Hawaii and Wellington are not shown. The city markers would overlap significantly if the entire world map were shown.
(4.33 MB MOV)

## Abbreviations

AR: attack rate
ECC: early combination chemotherapy
RAR: resistant attack rate
SMC: sequential multidrug chemotherapy
