# Supplementary material for: Hedging against Antiviral Resistance during the Next Influenza Pandemic Using Small Stockpiles of an Alternative Chemotherapy
Source: PLoS Med. 2009 May 19;6(5):e1000085. doi: 10.1371/journal.pmed.1000085 (PMC2680070; doi:10.1371/journal.pmed.1000085)
Supplement: Text S1 — Algorithms and additional sensitivity analyses. (3.66 MB PDF) [file pmed.1000085.s001.pdf]

Hedging against antiviral resistance during the next influenza pandemic using small stockpiles of an alternate chemotherapy

Joseph T Wu[*†], Gabriel M Leung[*], Marc Lipsitch[‡], Ben S. Cooper[§], Steven Riley[*]

[*] Department of Community Medicine and School of Public Health, Li Ka Shing Faculty of Medicine, The University of Hong Kong, Hong Kong SAR, China

[‡] Department of Epidemiology and Department of Immunology and Infectious Diseases, Harvard School of Public Health, 677 Huntington Avenue, Boston, MA 02115

[§] Statistics, Modelling, and Bioinformatics Department, Centre for Infections, Health Protection Agency, London, United Kingdom

[†] To whom correspondence should be addressed: joewu@hku.hk

Text S1

Algorithms 2
- Discrete-event stochastic simulation for a single population 2
- Discrete-time stochastic meta-population simulation for global network 4

Additional Sensitivity Analyses 9
- Reduction in infectiousness under combination chemotherapy 9
- Household-based antiviral prophylaxis 9
- The number of populations that implement antiviral intervention and SMC/ECC ..10
- When will SMC/ECC fail in downstream populations if the source does implement SMC/ECC? 11
- Zanamivir is an effective candidate for the secondary antiviral though it does not reduce infectiousness and is not licensed for use in small children 11
- Resistance emergence due to reassortment 12
- Deterministic Version of the Stochastic Model 13

References 15
Tables 17
Figures 20

Algorithms

Discrete-event stochastic simulation for a single population

Let $X^{(m,s,d)}(t)$ be the number of infected individuals who

1. Have been infected with strain s prior to time t;
2. Are in disease state m at time t;
3. Are taking drug regimen d at time t.

We call the combination (m,s,d) the status of the individual. The set of possible disease states of each individual is defined by the natural history model of influenza in Figure A, i.e. $m \in \{Exposed, Presymptomatic, Asymptomatic, Symptomatic\}$. We consider four possible strains:

1. Wild-type, which is sensitive to both drug A and B ($A_S B_S$);
2. Resistant to drug A but sensitive to drug B ($A_R B_S$);
3. Sensitive to drug A but resistant to drug B ($A_S B_R$);
4. Dually-resistant ($A_R B_R$).

We consider three drug regimens:

1. No antivirals ($None$);
2. Monotherapy with drug A ($MonoA$);
3. Monotherapy with drug B ($MonoB$)
4. Combination therapy with drug A and B ($Combo$).

The drug policy of the population may change over time as antivirals are depleted.

The transmission model is individual-based and the simulation is conducted in continuous time. Let N be the population size. The spread of disease is simulated by generating the time of the next infection and choosing the individual in whom that infection occurs. After infection, disease progression within an individual is determined by transition events such as "become symptomatic" as per the waiting time distributions defined in the natural history model. New infection events are generated by evaluating the instantaneous force of infection for each strain, drawing all waiting times from the appropriate exponential distributions and then simulating the time, individual, and strain associated with the next infection event. The instantaneous force of infection for strain s is

$$\lambda^s(t) = \frac{\beta}{N} \sum_{(m,d)} X_i^{(m,s,d)}(t) \cdot h(m,s,d), \qquad s \in \{A_S B_S, A_R B_S, A_S B_R, A_R B_R\},$$

where $h(m,s,d) = r(m) \cdot \delta(s,d)$ is the relative infectiousness of an infected individual with status (m,s,d),

$$r(m) = \begin{cases} 1 & \text{if } m = \mathit{Symptomatic}, \\ h_{Asymptomatic} & \text{if } m = \mathit{Asymptomatic} \\ h_{Presymptomatic} & \text{if } m = \mathit{Preymptomatic} \\ 0 & \text{otherwise,} \end{cases}$$

and

$$\delta(s,d) = \begin{cases} 1-\varepsilon & \begin{array}{l} \text{if strain } s \text{ was sensitive to at least} \\ \text{one of the antivirals in drug regimen } d, \end{array} \\ 1 & \text{otherwise.} \end{cases}$$

where ε is the reduction in infectiousness provided by antiviral treatment (see Figure B). β is calibrated to yield the desired R_0 using the formula

$$R_0 = \beta\left(h_{Presymptomatic} E\left[D_{Presymptomatic}\right] + (1-p_S) h_{Asymptomatic} E\left[D_{Asymptomatic}\right] + p_S E\left[D_{Symptomatic}\right]\right)$$

where $D_{Presymptomatic}$, $D_{Asymptomatic}$, and $D_{Symptomatic}$ are the durations of the presymptomatic, asymptomatic, and symptomatic stages .

To illustrate how we simulate emergence of resistance (see Figure B for the resistance emergence model), suppose the drug regimen is Mono throughout the pandemic. Upon onset of symptoms, an infected individual is treated with drug A with probability p_T. If the individual is treated, antiviral resistance emerges during his symptomatic stage with probability p_A. We assume that the rate of emergence within the individual r_A is constant throughout the symptomatic stage. As such, the time to emergence within an individual T_A is an exponential random variable with mean $1/r_A$. We compute r_A by solving the equation

$$1 - p_A = E\left[\exp\left(-r_A D_{Symptomatic}\right)\right]$$

where both sides of the equation represent the probability that resistance does not emerge. When an individual becomes symptomatic and is treated with drug A, $D_{Symptomatic}$ and T_A are drawn from their distributions and compared. If the latter is smaller, then resistance emerges T_A time units after treatment is initiated for the individual. Otherwise, the individual does not develop resistance. This model implicitly assumes that the hazard of emergence of resistance is constant over the infectious course of an individual once treatment begins, but results are weakly sensitive to this assumption since the overall probability of emergence p_A is fixed by assumption; hence the assumption of constant hazard affects only when, not whether, emergence of resistance occurs in a particular host. Although later emergence of resistance

in individuals should reduce the probability of transmission, it is unlikely that our conclusions are sensitive to our more simple model structure: a slight reduction in transmission from the initial resistant case due to the timing of its appearance must be dominated by the range of values over several orders of magnitude we use for de novo rates of emergence (p_A and p_B) in our sensitivity analysis.

Emergence of dual resistance within a single individual (i.e. $A_S B_S \to A_S B_R \to A_R B_R$ or $A_S B_S \to A_R B_S \to A_R B_R$) under combination chemotherapy is simulated using the same mechanism.

Discrete-time stochastic meta-population simulation for global network

The discrete-event simulation above is an exact algorithm for simulating the stochastic epidemic dynamics in a single closed population. However, the computational requirement of this method becomes prohibitive when applied to the many connected populations. We use a discrete-time simulation as an alternative, which is computationally more efficient at this scale. A time-step of 0.25 days is chosen for the discrete-time simulation. With this time-step, the results given by the discrete-time simulation and its discrete-event counterpart are statistically indistinguishable when simulating the pandemic in a single population.

Let N_i be the size of population i. Let $S_{ij}(t)$ be the number of residents of population i who are susceptible and are traveling in population j at time t. Let $R_{ij}^{s}(t)$ be the number of residents of population i who have recovered from a strain s infection and are traveling in population j at time t. Let $X_{ij}^{(m,s,d,\tau)}(t)$ be the number of individuals who

1. Are residents of population i;
2. Are infected with strain s at time t;
3. Are in disease state m at time t;
4. Are taking drug regimen d at time t;
5. Are traveling in population j at time t;
6. Will progress to the next disease state at time $t+\tau$.

We say that these individuals have status (m,s,d,τ,i,j) at time t. Since the simulation proceeds with time-step Δt, both t and τ are integer multiples of Δt.

The algorithm

To simulate the global spread of resistance, we first specify the initial conditions at time $t=0$. For example, if Hong Kong (with population index H) is the first city infected (the "source") with y initial infections with wild-type virus, then

$$
\begin{aligned}
&S_{HH}(0) = N_H - y, \\
&X_{HH}^{(Exposed, A_S B_S, None, \tau)}(0) = Exposed_\tau\left[y\right], \\
&S_{ii}(0) = N_i \quad \text{for all } i \neq H, \\
&\text{all other state variables are 0,}
\end{aligned}
$$

where $Exposed_\tau\left[y\right]$ is the number of newly infected individuals that have exposed duration τ given that there are y new infections. The random variable $Exposed_\tau\left[y\right]$ is generated from the latent duration distribution specified in the natural history model (see Figure A).

After the initial conditions have been specified, Steps 1 to 4 below are then executed for each time $t_k = k\Delta t$, $k = 1, 2, \ldots$ until $t_k = 365$ days:

Step 1: System states from the previous time step

The state variables at time t_k are initiated with the state variables from the previous time step:

$$
\begin{aligned}
&S_{ij}(t_k) \leftarrow S_{ij}(t_{k-1}), \\
&X_{ij}^{(m,s,d,\tau)}(t_k) \leftarrow X_{ij}^{(m,s,d,\tau)}(t_{k-1}), \\
&R_{ij}^{s}(t_k) \leftarrow R_{ij}^{s}(t_{k-1}).
\end{aligned}
$$

Step 2: Inter-population travel

Data from the International Air Transport Association for 2002 is used to construct an air travel volume matrix $W = \left\{w_{ij}\right\}$, where w_{ij} represents the rate at which a resident from population i travels to population j. An individual who travels to a foreign population returns to his home population after T_R days. We assume that T_R is exponentially distributed with mean 7 days. We illustrate below how inter-population travel is implemented for the susceptibles. The mechanism is exactly the same for all individuals.

Define the following random variables:

$Travel_{i\to j}\left[S_{ii}(t_k)\right]$: The number of susceptible residents of population i who travel to population j between time t_{k-1} and t_k.

$Return[S_{ij}(t_k)]$: The number of susceptible residents of population i who return home from population j between time t_{k-1} and t_k.

These random variables are distributed as follows:

$$Travel_{i\to j}\left[S_{ii}(t_k)\right] \sim Binomial(S_{ii}(t_k), 1-e^{-w_{ij}\Delta t}),$$
$$Return\left[S_{ij}(t_k)\right] \sim Binomial(S_{ij}(t_k), 1-e^{-\Delta t/T_R}).$$

Suppose the realized values are $Travel_{i\to j}\left[S_{ii}(t_k)\right] = z_{ij}$ and $Return[S_{ij}(t_k)] = u_{ij}$. Then the $S_{ij}(t_k)$'s are updated as follows:

$$S_{ii}(t_k) \leftarrow S_{ii}(t_k) - \sum_j z_{ij} + \sum_j u_{ij},$$
$$S_{ij}(t_k) \leftarrow S_{ij}(t_k) + z_{ij} - u_{ij} \quad \text{for } i \neq j.$$

Step 3: Emergence of resistance

Let $\alpha(\cdot)$ be the function that maps emergence probabilities to emergence rates (e.g., $r_A = \alpha(p_A)$). Let $emergence\left[X_{ij}^{(Symptomatic,s,d,\tau)}(t_k)\right]$ be the number of individuals with status $(Symptomatic, s, d, \tau, i, j)$ who develop antiviral resistance between time t_{k-1} and t_k. The possible paths of resistance emergence and the associated probability distributions are as follows:

1. $(Symptomatic, A_S B_S, MonoA, \tau, i, j) \to (Symptomatic, A_R B_S, MonoA, \tau, i, j)$:
$$emergence\left[X_{ij}^{(Symptomatic,A_S B_S,MonoA,\tau)}(t_k)\right] \sim Binomial\left(X_{ij}^{(Symptomatic,A_S B_S,MonoA,\tau)}(t_k), 1-e^{-\alpha(p_A)\Delta t}\right)$$

2. $(Symptomatic, A_S B_S, MonoB, \tau, i, j) \to (Symptomatic, A_S B_R, MonoB, \tau, i, j)$:
$$emergence\left[X_{ij}^{(Symptomatic,A_S B_S,MonoB,\tau)}(t_k)\right] \sim Binomial\left(X_{ij}^{(Symptomatic,A_S B_S,MonoB,\tau)}(t_k), 1-e^{-\alpha(p_B)\Delta t}\right)$$

3. $(Symptomatic, A_S B_S, Combo, \tau, i, j) \to (Symptomatic, A_R B_S, Combo, \tau, i, j)$ or $(Symptomatic, A_S B_S, Combo, \tau, i, j) \to (Symptomatic, A_S B_R, Combo, \tau, i, j)$:
$$emergence\left[X_{ij}^{(Symptomatic,A_S B_S,Combo,\tau)}(t_k)\right] \sim Binomial\left(X_{ij}^{(Symptomatic,A_S B_S,Combo,\tau)}(t_k), 1-e^{-(\alpha((1-s)p_A)+\alpha((1-s)p_B))\Delta t}\right)$$
If $emergence\left[X_{ij}^{(Symptomatic,A_S B_S,Combo,\tau)}(t_k)\right] = n$, then $Binomial\left(n, \dfrac{\alpha((1-s)p_A)}{\alpha((1-s)p_A)+\alpha((1-s)p_B)}\right)$ of them are $A_R B_S$ while the rest are $A_S B_R$.

4. $(Symptomatic, A_S B_R, MonoA, \tau, i, j) \to (Symptomatic, A_R B_R, MonoA, \tau, i, j)$:
$$emergence\left[X_{ij}^{(Symptomatic,A_S B_R,MonoA,\tau)}(t)\right] \sim Binomial\left(X_{ij}^{(Symptomatic,A_S B_R,MonoA,\tau)}(t), 1-e^{-\alpha(p_A)\Delta t}\right)$$

5. $(Symptomatic, A_S B_R, MonoA, \tau, i, j) \to (Symptomatic, A_R B_R, MonoA, \tau, i, j)$:
$$emergence\left[X_{ij}^{(Symptomatic,A_R B_S,MonoB,\tau)}(t)\right] \sim Binomial\left(X_{ij}^{(Symptomatic,A_R B_S,MonoB,\tau)}(t), 1-e^{-\alpha(p_B)\Delta t}\right)$$

6. $(Symptomatic, A_S B_R, Combo, \tau, i, j) \to (Symptomatic, A_R B_R, Combo, \tau, i, j)$:
$$emergence\left[X_{ij}^{(Symptomatic,A_S B_R,Combo,\tau)}(t)\right] \sim Binomial\left(X_{ij}^{(Symptomatic,A_S B_R,Combo,\tau)}(t), 1-e^{-\alpha(p_A)\Delta t}\right)$$

7. $(Symptomatic, A_R B_S, Combo, \tau, i, j) \to (Symptomatic, A_R B_R, Combo, \tau, i, j)$:
$$emergence\left[X_{ij}^{(Symptomatic,A_R B_S,Combo,\tau)}(t)\right] \sim Binomial\left(X_{ij}^{(Symptomatic,A_R B_S,Combo,\tau)}(t), 1-e^{-\alpha(p_B)\Delta t}\right)$$

The state variables $X_{ij}^{(Symptomatic,s,d,\tau)}(t_k)$ are updated accordingly after these emergence events are generated.

Step 4: Infection and disease progression

Infection. The instantaneous force of infection for strain s in population i at time t_k is

$$\lambda_i^s(t_k) = \frac{\beta}{N_i} \sum_{(m,d,\tau,j)} X_{ji}^{(m,s,d,\tau)}(t_k) \cdot h(m,s,d), \qquad s \in \{A_S B_S, A_R B_S, A_S B_R, A_R B_R\}.$$

Let $Infection_j^s\left[S_{ij}(t_k)\right]$ be the number of strain s infections among residents of population i who are in population j at time t_k. These infection events are generated with the averaged force of infection $\left(\lambda_j^s(t_k) + \lambda_j^s(t_{k-1})\right)/2$:

$$Infection_s\left[S_{ij}(t_k)\right] \sim Binomial\left(S_{ij}(t_k), 1 - e^{-\Delta t(\lambda_j^s(t_k)+\lambda_j^s(t_{k-1}))/2}\right)$$

Disease progression. The $X_{ij}^{(Presymptomatic,s,None,\Delta t)}(t_k)$ presymptomatic individuals with status $(Presymptomatic, s, None, \Delta t, i, j)$ progress to the next disease stage. Upon exiting the presymptomatic stage, these infected individual can become asymptomatic, symptomatic and untreated, or symptomatic and treated. Let A_{ij}^s, U_{ij}^s, and T_{ij}^s be the number of individuals with status $(Presymptomatic, s, None, \Delta t, i, j)$ who become asymptomatic, untreated symptomatic, and treated symptomatic at time t_k. The random variables $(A_{ij}^s, U_{ij}^s, T_{ij}^s)$ are drawn from a multinomial distribution with $X_{ij}^{(Presymptomatic,s,None,\Delta t)}(t_k)$ trials and probabilities $(1-p_S, p_S(1-p_T), p_S p_T)$, where $p_T = 0$ if there is no antiviral intervention at time t_k.

Suppose the realized values are $Infection_s\left[S_{ij}(t_k)\right] = v_{ij}^s$, $A_{ij}^s = a_{ij}^s$, $U_{ij}^s = u_{ij}^s$, and $T_{ij}^s = t_{ij}^s$. Then the following updates are performed:

$$S_{ij}(t_k) \leftarrow S_{ij}(t_k) - v_{ij}^{s},$$

$$X_{ij}^{(Exposed,s,None,\tau)}(t_k) \leftarrow X_{ij}^{(Exposed,s,None,\tau+\Delta t)}(t_k) + Exposed_{\tau}\left[v_{ij}^{s}\right],$$

$$X_{ij}^{(Presymptomatic,s,None,\tau)}(t_k) \leftarrow X_{ij}^{(Presymptomatic,s,None,\tau+\Delta t)}(t_k) + Presymptomatic_{\tau}\left[X_{ij}^{(Exposed,s,None,\Delta t)}\right]$$

$$X_{ij}^{(Asymptomatic,s,None,\tau)}(t_k) \leftarrow X_{ij}^{(Asymptomatic,s,None,\tau+\Delta t)}(t_k) + Asymptomatic_{\tau}\left[a_{ij}^{s}\right],$$

$$X_{ij}^{(Symptomatic,s,None,\tau)}(t_k) \leftarrow X_{ij}^{(Symptomatic,s,None,\tau+\Delta t)}(t_k) + Symptomatic_{\tau}\left[u_{ij}^{s}\right],$$

$$X_{ij}^{(Symptomatic,s,DP_i(t_k),\tau)}(t_k) \leftarrow X_{ij}^{(Symptomatic,s,DP_i(t_k),\tau+\Delta t)}(t_k) + Symptomatic_{\tau}\left[t_{ij}^{s}\right] \text{ if } DP_i(t_k) \neq None,$$

$$X_{ij}^{(Symptomatic,s,d,\tau)}(t_k) \leftarrow X_{ij}^{(Symptomatic,s,d,\tau+\Delta t)}(t_k) \text{ if } d \neq None \text{ and } d \neq DP_i(t_k),$$

$$R_{ij}^{s}(t_k) \leftarrow R_{ij}^{s}(t_k) + \sum_{d} X_{ij}^{(Symptomatic,s,d,\Delta t)}(t_k) + X_{ij}^{(Asymptomatic,s,None,\Delta t)}(t_k),$$

where $DP_i(t_k)$ is the drug policy of population i at time t_k, and the operators $Presymptomatic_{\tau}[\cdot]$, $Asymptomatic_{\tau}[\cdot]$ and $Symptomatic_{\tau}[\cdot]$ are analogous to $Exposed_{\tau}[\cdot]$ (defined in this section before Step 1).

Additional Sensitivity Analyses

Reduction in infectiousness under combination chemotherapy

In the main text, we assumed that if a symptomatic individual infected with strain s was treated with antiviral regimen d, his infectiousness was reduced by a proportion $\varepsilon = 0.67$ if strain s was sensitive to at least one drug in regimen d [1]. However, combination chemotherapy is likely to be more efficacious than monotherapy in reducing infectiousness [2]. If we set $\varepsilon = 1$ when a wild-type infection was treated with combination chemotherapy, results (not shown) were indistinguishable from those presented in the main text. Thus, our conclusions are not sensitive to higher values of ε under combination chemotherapy.

Household-based antiviral prophylaxis

In the main text, we assumed that large-scale antiviral intervention involved only treatment of symptomatic individuals. Recent studies on mitigation of influenza pandemics suggested that adding targeted antiviral prophylaxis could further reduce the attack rate [3,4] and many countries are now considering this option (http://www.hhs.gov/pandemicflu/plan/sup7.html). Here, we show that our conclusions in the main text hold when household-based antiviral prophylaxis is implemented in addition to treatment of symptomatic individuals. For illustration, we only present the results for ECC. Results for SMC are similar to that for ECC with high synergy.

To model household-based antiviral prophylaxis, we used an individual-based network simulation that we have previously built to study mitigation of influenza pandemics [4]. See Ref 4 for the details on model specifications. To make direct comparison with the results presented in the main text, we made the following modifications to the simulation:

1. The multi-strain structure and the process of resistance emergence were modeled as described in *Discrete-event stochastic simulation for a single population* above. Rates of resistance emergence during the presymptomatic and asymptomatic stages were the same as that during the symptomatic stage. Rates of resistance emergence were the same for both treatment and prophylaxis (a worst-case assumption).
2. The effect of hospitalization was ignored.
3. Household-based quarantine was not modeled.
4. Household-based antiviral prophylaxis was assumed to take place as follows. Upon onset of symptoms, an individual who was not already in the intervention program reported with probability $p_T = 0.4$ and received antiviral treatment. In addition, all household members of this individual were immediately recruited into the program and received antivirals: non-symptomatic members received prophylactic regimen for 5 days while symptomatic members (who did not report symptoms earlier) received treatment regimen. The “prophylactic clock” of 5 days

was renewed whenever a previously non-symptomatic household member became symptomatic.

5. Antiviral prophylaxis reduced susceptibility by 85% [1].
6. A 1% stockpile coverage of drug B was available for ECC (as in the main text).

Figure D-A shows that this network model (with household- and workplace-structures) and the simpler homogeneous mixing model in the main text gave essentially the same predictions on AR and RAR when antiviral intervention involved only treatment of symptomatic individuals. Therefore, household- and workplace-structures had little effect on the emergence and spread of resistance. When household-based antiviral prophylaxis was added, AR actually increased unless $p_A < 10^{-3}$. This is because the addition of prophylaxis required a higher level of antiviral use (approximately tripling the stockpile requirement) which speeded up the emergence of antiviral resistance at the population level.

Figure D-B is analogous to Figure1D with household-based antiviral prophylaxis added. The reductions in AR and RAR by ECC were smaller than that in Figure 1D. This was because when prophylaxis was implemented, antivirals were depleted faster. As a result, the wild-type prevalence was smaller (compared to the treatment-only scenario) when the 1 % stockpile coverage of drug B was used up, and therefore the subsequent emergence and spread of resistance was more severe. To restore the same level of effectiveness of ECC (as in Figure 1D), the stockpile of drug B needed to be increased by the same proportion of increase in drug A required by prophylaxis (compared to treatment only). Since the stockpile of drug A needed to be approximately tripled when household-based prophylaxis was added, the reductions in AR and RAR were similar to that presented in the main text when the stockpile coverage of drug B was boosted to 3% (Figure D-C).

These results suggested that our conclusion in the main text remains valid when household-based antiviral prophylaxis is implemented in addition to treatment.

The number of populations that implement antiviral intervention and SMC/ECC

In the main text, we assumed that 28 out of 105 cities in the global network implement antiviral intervention with Hong Kong as the source. To show that the conclusions made in the main text are robust against this assumption, we varied the number of populations that implement antiviral intervention, M, from 8 to 64. In Figure G, we simulated the scenarios in Figure 3A-D for each value of M. For scenario C and D (as in Figure 3), we also varied the proportion of the M populations that implement SMC (which we denoted by q) from 0.25 to 0.75. The policies adopted by Hong Kong, London, New York and Geneva in each scenario are the same as in Figure 3. In each realization, the source population was chosen randomly from the network (subject to the

exception that (i) the source cannot be London in scenario c because London implements monotherapy, and (ii) the source cannot be Hong Kong, New York and Geneva in scenario d because these populations implement SMC) Figure G shows that the conclusions drawn from Figure 3 are applicable in all scenarios regardless of the values of M and q.

When will SMC/ECC fail in downstream populations if the source does implement SMC/ECC?

In the main text, we claimed that if the source population did implement SMC, SMC became much less effective in downstream populations only if most of the neighbors (i.e. those populations that were immediately downstream) of the source failed to control resistance, which was likely only if most of these neighbors implemented only monotherapy and p_A was high. Here, we provide results to support this claim. We considered the same scenarios as in Figure 3A-C but with Kinshasa as the source of infection in the network with 30 wild-type seeds on day 0. Kinshasa was chosen as the source because it has the fewest neighbors in our network model (six neighbors, which are Addis Ababa, Brussels, Johannesburg, Lagos, Nairobi, and Paris). In each scenario, we increased the number of populations that implement monotherapy by n, where $n = 0, 2, 4, 6$. We conducted two sets of simulations: (a) the n additional monotherapy populations were chosen randomly from the network; (b) the n additional monotherapy populations were chosen randomly from the neighbors of Kinshasa. Figure H shows that for a given p_A, the spread of resistance was essentially insensitive to the value of n in Set (a) (which is consistent with the results in Figure G) but increased considerably with n in Set (b). In Set (b), the effectiveness of SMC diminished significantly only when n was large (relatively to the total number of neighbors) and p_A was larger than 0.05. Similar results were obtained for a different source population. Taken together, these results give support to our claim that SMC becomes much less effective in downstream populations only if most of the neighbors of the source fail to control resistance, which is unlikely unless most these neighbors implement only monotherapy and p_A is very high.

Zanamivir is an effective candidate for the secondary antiviral though it does not reduce infectiousness and is not licensed for use in small children

In the main text, we claimed that although zanamivir is not licensed for treatment in children less than 7 years of age and may not be able to reduce infectiousness, these drawbacks have little impact on the effectiveness of SMC and ECC. We justify this claim in this section.

Empirical evidence suggested that zanamivir provides no reduction in infectiousness [5]. However, our results have shown that reduction in infectiousness by the secondary antiviral is not required for SMC or ECC to be an effective hedge: if the secondary antiviral provides no reduction in infectiousness, then SMC is the same as delaying large-scale antiviral

intervention, which we have shown to be an effective hedge (Figure 1C). For ECC, we have assumed that the reduction in infectiousness under combination chemotherapy is the same as that provided by the primary antiviral alone ($\varepsilon = 66\%$), which would be the case if the secondary antiviral provides no reduction in infectiousness and does not interfere with the action of the primary antiviral. Therefore, zanamivir should not be ruled out as a suitable secondary antiviral on the basis of a potential lack of reduction in infectiousness.

Another potential problem with using zanamivir as the secondary antiviral is that zanamivir is not licensed for treatment in children less than 7 years of age. However, the need for treating this special group with the primary antiviral (oseltamivir) has little impact on the overall effectiveness of SMC and ECC. For instance, this group accounts for ~10% of the population in the US and treating this group with the primary drug under SMC is similar to reducing p_A by 10 times at the population level during the early phase of large-scale antiviral intervention. Further, SMC and ECC will still reduce the spread of A-resistance during the early phase by lowering the infectiousness of treated individuals who do not belong to the special group and are infected with an A-resistant strain that is not dually resistant. These effects together ensure that the overall effectiveness of SMC and ECC will only be slightly attenuated by the need for treating the special group with the primary antiviral.

Resistance emergence due to reassortment

Recent establishment of the oseltamivir-resistant H1N1 strain [6,7,8,9,10,11,12] has elevated the concern that an oseltamivir-sensitive pandemic strain may gain resistance via reassortment with circulating oseltamivir-resistant strains. We have shown in Figure 1C that the spread of resistance is limited if the cumulative number of infections at the time resistance emerges (in a population of 6.8 million) is 100,000 or above. Therefore, as long as the probability of resistance emergence via any means other than drug pressure is less than 0.00001 for every infected case, our conclusions in this study will hold. To put this threshold in the context of reassortment with seasonal strains, consider a region where the epidemic curve of seasonal influenza is relatively flat with an annual attack rate of 15% (e.g. Singapore). For resistance to emerge via reassortment between the pandemic strain and a circulating seasonal strain, an individual must be coinfected by both strains. Assume that the pandemic has no effect on the transmission of seasonal influenza, which is a conservative assumption favoring reassortment. In this scenario, if an individual is infected with the pandemic strain, the probability that he will be coinfected by a seasonal strain during his pandemic infection is on the order of 0.001 (0.15/365*duration of pandemic infection in days). If we multiply this by the probability of the occurrence of a reassortment event that gives rise to a resistant and fully fit strain in a coinfected individual (say, 1 in 100), the contribution of reassortment to resistance emergence will likely satisfy the 0.00001-threshold and is small compared to drug-induced resistance in our base case. Figure K confirms this.

Deterministic Version of the Stochastic Model

The modeling equations for the deterministic model are shown below:

	$Policy(t) = MonoA$	$Policy(t) = MonoB$	$Policy(t) = Combo$
Susceptible	$\frac{dS}{dt} = -\left(\lambda^{A_S B_S}(t) + \lambda^{A_R B_S}(t) + \lambda^{A_S B_R}(t) + \lambda^{A_R B_R}(t)\right)S,$<br>$\lambda^{A_S B_S}(t) = \frac{\beta}{N}\left(h_{Presymptomatic}P_{A_S B_S} + h_{Asymptomatic}A_{A_S B_S} + I_{A_S B_S,None} + (1-\varepsilon)(I_{A_S B_S,MonoA} + I_{A_S B_S,MonoB} + I_{A_S B_S,Combo})\right),$<br>$\lambda^{A_R B_S}(t) = \frac{\beta}{N}\left(h_{Presymptomatic}P_{A_R B_S} + h_{Asymptomatic}A_{A_R B_S} + I_{A_R B_S,None} + I_{A_R B_S,MonoA} + (1-\varepsilon)(I_{A_R B_S,MonoB} + I_{A_R B_S,Combo})\right),$<br>$\lambda^{A_S B_R}(t) = \frac{\beta}{N}\left(h_{Presymptomatic}P_{A_S B_R} + h_{Asymptomatic}A_{A_S B_R} + I_{A_S B_R,None} + I_{A_S B_R,MonoB} + (1-\varepsilon)(I_{A_S B_R,MonoA} + I_{A_S B_R,Combo})\right),$<br>$\lambda^{A_R B_R}(t) = \frac{\beta}{N}\left(h_{Presymptomatic}P_{A_R B_R} + h_{Asymptomatic}A_{A_R B_R} + I_{A_R B_R,None} + I_{A_R B_R,MonoA} + I_{A_R B_R,MonoB} + I_{A_R B_R,Combo}\right)$		
Exposed	$\frac{dE_s}{dt} = \lambda^s(t)S - \frac{E_s}{D_{Exposed}} \quad \forall s$		
Presymptomatic	$\frac{dP_s}{dt} = \frac{E_s}{D_{Exposed}} - \frac{P_s}{D_{Presymptomatic}} \quad \forall s$		
Asymptomatic	$\frac{dA_s}{dt} = (1-p_S)\frac{P_s}{D_{Presymptomatic}} - \frac{A_s}{D_{Asymptomatic}} \quad \forall s$		
Symptomatic	$\frac{dI_{s,None}}{dt} = (1-p_T)\,p_S\frac{P_s}{D_{Presymptomatic}} - \frac{I_{i,None}}{D_{Symptomatic}} \forall s,$<br>$\frac{dI_{A_S B_S,MonoA}}{dt} = (1-p_A)\,p_T p_S\frac{P_{A_S B_S}}{D_{Presymptomatic}} - \frac{I_{A_S B_S,MonoA}}{D_{Symptomatic}},$<br>$\frac{dI_{A_R B_S,MonoA}}{dt} = p_T p_S\frac{P_{A_R B_S}}{D_{Presymptomatic}} + p_A p_T p_S\frac{P_{A_S B_S}}{D_{Presymptomatic}} - \frac{I_{A_R B_S,MonoA}}{D_{Symptomatic}},$<br>$\frac{dI_{A_S B_R,MonoA}}{dt} = (1-p_A)\,p_T p_S\frac{P_{A_S B_R}}{D_{Presymptomatic}} - \frac{I_{A_S B_R,MonoA}}{D_{Symptomatic}},$<br>$\frac{dI_{A_R B_R,MonoA}}{dt} = p_T p_S\frac{P_{A_R B_R}}{D_{Presymptomatic}} + p_A p_T p_S\frac{P_{A_S B_R}}{D_{Presymptomatic}} - \frac{I_{A_R B_R,MonoA}}{D_{Symptomatic}},$<br>$\frac{dI_{s,d}}{dt} = -\frac{I_{s,d}}{D_{Symptomatic}} \quad \forall s,\ \ d \ne None$ and $d \ne MonoA$	$\frac{dI_{s,None}}{dt} = (1-p_T)\,p_S\frac{P_s}{D_{Presymptomatic}} - \frac{I_{s,None}}{D_{Symptomatic}} \forall s,$<br>$\frac{dI_{A_S B_S,MonoB}}{dt} = (1-p_B)\,p_T p_S\frac{P_{A_S B_S}}{D_{Presymptomatic}} - \frac{I_{A_S B_S,MonoB}}{D_{Symptomatic}},$<br>$\frac{dI_{A_R B_S,MonoB}}{dt} = (1-p_B)\,p_T p_S\frac{P_{A_R B_S}}{D_{Presymptomatic}} - \frac{I_{A_R B_S,MonoB}}{D_{Symptomatic}},$<br>$\frac{dI_{A_S B_R,MonoB}}{dt} = p_T p_S\frac{P_{A_S B_R}}{D_{Presymptomatic}} + p_B p_T p_S\frac{P_{A_S B_S}}{D_{Presymptomatic}} - \frac{I_{A_S B_R,MonoB}}{D_{Symptomatic}},$<br>$\frac{dI_{A_R B_R,MonoB}}{dt} = p_T p_S\frac{P_{A_R B_R}}{D_{Presymptomatic}} + p_B p_T p_S\frac{P_{A_R B_S}}{D_{Presymptomatic}} - \frac{I_{A_R B_R,MonoB}}{D_{Symptomatic}},$<br>$\frac{dI_{s,d}}{dt} = -\frac{I_{s,d}}{D_{Symptomatic}} \quad \forall s,\ \ d \ne None$ and $d \ne MonoB$	$\frac{dI_{s,None}}{dt} = (1-p_T)\,p_S\frac{P_s}{D_{Presymptomatic}} - \frac{I_{s,None}}{D_{Symptomatic}} \quad \forall s,$<br>$\frac{dI_{A_S B_S,Combo}}{dt} = \left(1-(1-s)(p_A+p_B)\right)p_T p_S\frac{P_{A_S B_S}}{D_{Presymptomatic}} - \frac{I_{A_S B_S,Combo}}{D_{Symptomatic}},$<br>$\frac{dI_{A_R B_S,Combo}}{dt} = (1-p_B)\,p_T p_S\frac{P_{A_R B_S}}{D_{Presymptomatic}} + (1-s)\,p_A p_T p_S\frac{P_{A_S B_S}}{D_{Presymptomatic}} - \frac{I_{A_R B_S,Combo}}{D_{Symptomatic}},$<br>$\frac{dI_{A_S B_R,Combo}}{dt} = (1-p_A)\,p_T p_S\frac{P_{A_S B_R}}{D_{Presymptomatic}} + (1-s)\,p_B p_T p_S\frac{P_{A_S B_S}}{D_{Presymptomatic}} - \frac{I_{A_S B_R,Combo}}{D_{Symptomatic}},$<br>$\frac{dI_{A_R B_R,Combo}}{dt} = p_T p_S\frac{P_{A_R B_R}}{D_{Presymptomatic}} + p_A p_T p_S\frac{P_{A_S B_R}}{D_{Presymptomatic}} + p_B p_T p_S\frac{P_{A_R B_S}}{D_{Presymptomatic}} - \frac{I_{A_R B_R,Combo}}{D_{Symptomatic}},$<br>$\frac{dI_{s,d}}{dt} = -\frac{I_{s,d}}{D_{Symptomatic}} \quad \forall s,\ d \ne None$ and $d \ne Combo$
Removed	$\frac{dR_s}{dt} = \frac{A_s}{D_{Asymptomatic}} + \frac{I_{s,None}}{D_{Symptomatic}} + \frac{I_{s,MonoA}}{D_{Symptomatic}} + \frac{I_{s,MonoB}}{D_{Symptomatic}} + \frac{I_{s,Combo}}{D_{Symptomatic}} \quad \forall s$		
Drug A and B used	$\frac{dM_A}{dt} = p_T p_S\frac{P_{A_S B_S} + P_{A_R B_S} + P_{A_S B_R} + P_{A_R B_R}}{D_{Presymptomatic}},\ \ \frac{dM_B}{dt} = 0$	$\frac{dM_A}{dt} = 0,\ \ \frac{dM_B}{dt} = p_T p_S\frac{P_{A_S B_S} + P_{A_R B_S} + P_{A_S B_R} + P_{A_R B_R}}{D_{Presymptomatic}}$	$\frac{dM_A}{dt} = p_T p_S\frac{P_{A_S B_S} + P_{A_R B_S} + P_{A_S B_R} + P_{A_R B_R}}{D_{Presymptomatic}},\ \ \frac{dM_B}{dt} = p_T p_S\frac{P_{A_S B_S} + P_{A_R B_S} + P_{A_S B_R} + P_{A_R B_R}}{D_{Presymptomatic}}$

The state variables are defined as follows:

S : Susceptible
E_s : Exposed individuals infected by strain s
P_s : Presymptomatic individuals infected by strain s
A_s : Asymptomatic individuals infected by strain s
$I_{s,d}$: Symptomatic individuals infected by strain s and treated with drug regimen d
R_s : Removed individuals who were infected by strain s
M_A : Amount of drug A used (in terms of population coverage, i.e. number of treatment courses per capita)
M_B : Amount of drug B used

$Policy(t)$ is the drug regimen used for large-scale antiviral intervention at time t and is defined as follows:

Under monotherapy,

$$Policy(t) = MonoA \text{ for all t.}$$

Under SMC:

$$Policy(t) = \begin{cases} MonoB \text{ if } M_B(t) < 0.01, \\ MonoA \text{ otherwise.} \end{cases}$$

Under ECC:

$$Policy(t) = \begin{cases} Combo \text{ if } M_B(t) < 0.01, \\ MonoA \text{ otherwise.} \end{cases}$$

All parameters and symbols in the above system have been defined earlier in the section “Algorithms” (except that the mean duration for disease stage i is denoted here by D_i instead of $E[D_i]$).

References

1. Lipsitch M, Cohen T, Murray M, Levin BR (2007) Antiviral resistance and the control of pandemic influenza. PLoS Med 4: e15.
2. Ilyushina NA, Hoffmann E, Salomon R, Webster RG, Govorkova EA (2007) Amantadine-oseltamivir combination therapy for H5N1 influenza virus infection in mice. Antivir Ther 12: 363-370.
3. Germann TC, Kadau K, Longini IM, Jr., Macken CA (2006) Mitigation strategies for pandemic influenza in the United States. Proc Natl Acad Sci U S A 103: 5935-5940.
4. Wu JT, Riley S, Fraser C, Leung GM (2006) Reducing the impact of the next influenza pandemic using household-based public health interventions. PLoS Med 3: e361.
5. Halloran ME, Hayden FG, Yang Y, Longini IM, Jr., Monto AS (2007) Antiviral effects on influenza viral transmission and pathogenicity: observations from household-based trials. Am J Epidemiol 165: 212-221.
6. (2008) Update: influenza activity--United States, September 30, 2007-February 9, 2008. MMWR Morb Mortal Wkly Rep 57: 179-183.
7. Dharan NJ, Gubareva LV, Meyer JJ, Okomo-Adhiambo M, McClinton RC, et al. (2009) Infections with oseltamivir-resistant influenza A(H1N1) virus in the United States. Jama 301: 1034-1041.
8. Hauge SH, Dudman S, Borgen K, Lackenby A, Hungnes O (2009) Oseltamivir-resistant influenza viruses A (H1N1), Norway, 2007-08. Emerg Infect Dis 15: 155-162.
9. Meijer A, Lackenby A, Hungnes O, Lina B, van-der-Werf S, et al. (2009) Oseltamivir-resistant influenza virus A (H1N1), Europe, 2007-08 Season. Emerg Infect Dis 15: 552-560.
10. Moscona A (2009) Global transmission of oseltamivir-resistant influenza. N Engl J Med 360: 953-956.
11. Sheu TG, Deyde VM, Okomo-Adhiambo M, Garten R, Xu X, et al. (2008) Surveillance for neuraminidase inhibitor resistance among human influenza A and B viruses circulating worldwide in 2004-2008. Antimicrob Agents Chemother.
12. Tamura D, Mitamura K, Yamazaki M, Fujino M, Nirasawa M, et al. (2009) Oseltamivir-Resistant Influenza A Viruses Circulating in Japan. J Clin Microbiol.
13. Ferguson NM, Cummings DA, Fraser C, Cajka JC, Cooley PC, et al. (2006) Strategies for mitigating an influenza pandemic. Nature 442: 448-452.
14. Longini IM, Jr., Nizam A, Xu S, Ungchusak K, Hanshaoworakul W, et al. (2005) Containing pandemic influenza at the source. Science 309: 1083-1087.
15. Ferguson NM, Cummings DA, Cauchemez S, Fraser C, Riley S, et al. (2005) Strategies for containing an emerging influenza pandemic in Southeast Asia. Nature 437: 209-214.
16. Hayden FG, Tunkel AR, Treanor JJ, Betts RF, Allerheiligen S, et al. (1994) Oral LY217896 for prevention of experimental influenza A virus infection and illness in humans. Antimicrob Agents Chemother 38: 1178-1181.

17. Viboud C, Boelle PY, Cauchemez S, Lavenu A, Valleron AJ, et al. (2004) Risk factors of influenza transmission in households. Br J Gen Pract 54: 684-689.
18. Fraser C, Riley S, Anderson RM, Ferguson NM (2004) Factors that make an infectious disease outbreak controllable. Proc Natl Acad Sci U S A 101: 6146-6151.

Tables

Table A. Assumptions for key unknown transmission parameters. Ranges presented here were used for sensitivity analyses. Parameters were translated into symptomatic and asymptomatic durations and relative infectiousness for different disease stages (see Figure A).

Parameter	Baseline Values	Range for sensitivity analyses	Notes / References
R_0, the average number of secondary cases generated by a typically infectious individual in an otherwise susceptible population	1.8	[1, 3]	Refs 3, 4 and 13-15
θ, the proportion of transmission by people who are not symptomatic (either presymptomatic or asymptomatic)	0.3	[0, 0.3]	Broad sensitivity analysis. Baseline value at upper bound to avoid overestimating the effectiveness of antiviral intervention.
T_g, the average time between the infection of an infector and the infection of his or her infectees	2.6	[2, 4]	Baseline value from Ref 13. Absolute durations of symptomatic and asymptomatic infectious stages derived from this parameter.
Proportion of infections counted in θ by those who are never symptomatic	0.5	[0.33,1]	Broad sensitivity analysis. Lower bound must be greater than proportion of cases asymptomatic.

Table B. Attack rates and resistant attack rates under SMC and ECC. Three probabilities of resistance emergence for drug B are shown here: p_B = 0.01 (upper table), 0.05 (middle table), and 0.3 (lower table).

p_A	Attack rate (%)				Resistant attack rate (%)			
	**Mono**	**SMC**	**ECC0**	**ECC1**	**Mono**	**SMC**	**ECC0**	**ECC1**
0.001	58 (56, 68)	56 (56, 57)	56 (56, 57)	56 (56, 56)	8 (1, 43)	0 (0, 0)	1 (0, 4)	0 (0, 0)
0.01	67 (62, 72)	57 (57, 58)	59 (58, 64)	57 (57, 57)	37 (18, 64)	2 (2, 3)	9 (5, 24)	3 (3, 3)
0.1	72 (71, 73)	63 (63, 63)	67 (66, 71)	63 (63, 63)	66 (60, 71)	17 (15, 18)	39 (32, 57)	18 (18, 18)

p_A	Attack rate (%)				Resistant attack rate (%)			
	**Mono**	**SMC**	**ECC0**	**ECC1**	**Mono**	**SMC**	**ECC0**	**ECC1**
0.001	58 (56, 68)	57 (56, 57)	56 (56, 60)	56 (56, 56)	8 (1, 43)	0 (0, 0)	2 (0, 11)	0 (0, 0)
0.01	67 (62, 72)	58 (57, 58)	61 (58, 68)	57 (57, 57)	37 (18, 64)	2 (2, 2)	14 (6, 43)	3 (3, 3)
0.1	72 (71, 73)	63 (63, 63)	69 (67, 72)	63 (63, 63)	66 (60, 71)	15 (15, 16)	49 (38, 65)	18 (18, 18)

p_A	Attack rate (%)				Resistant attack rate (%)			
	**Mono**	**SMC**	**ECC0**	**ECC1**	**Mono**	**SMC**	**ECC0**	**ECC1**
0.001	58 (56, 68)	57 (57, 57)	58 (56, 66)	56 (56, 56)	8 (1, 43)	0 (0, 0)	5 (1, 33)	0 (0, 0)
0.01	67 (62, 72)	58 (58, 58)	65 (60, 71)	57 (57, 57)	37 (18, 64)	2 (2, 2)	29 (12, 59)	3 (3, 3)
0.1	72 (71, 73)	63 (63, 63)	72 (70, 73)	63 (63, 63)	66 (60, 71)	14 (14, 15)	62 (54, 69)	18 (18, 18)

Table C. Illustrative example attack rates (AR) and resistant attack rates (RAR) under sequential multi-drug chemotherapy (SMC) and early combination chemotherapy (ECC) with synergy of 0 (ECC0) or 1 (ECC1) using the deterministic model.

Attack rate and resistant attack rate (%)						
p_A	Mono	SMC	ECC1	ECC0 with $p_B = 0.01$	ECC0 with $p_B = 0.05$	ECC0 with $p_B = 0.3$
0.001	64<br>24	57<br>0	56<br>0	56<br>2	57<br>3	59<br>10
0.01	70<br>54	58<br>3	57<br>4	60<br>12	62<br>20	67<br>37
0.1	73<br>69	64<br>20	64<br>22	69<br>45	70<br>53	72<br>63

Note: In each cell, the attack rate (upper row) and resistant attack rate (lower row) are shown. Three probabilities of resistance emergence for drug B are shown here: p_B = 0.01, 0.05, and 0.3. Under SMC and ECC1 (ECC with perfect synergy), AR and RAR are insensitive to the value of p_B in this range, hence only one set of outcomes is shown. Comparison with Table 1 shows that the deterministic model is not necessarily an accurate estimate of the mean behavior of the stochastic model.

Figures

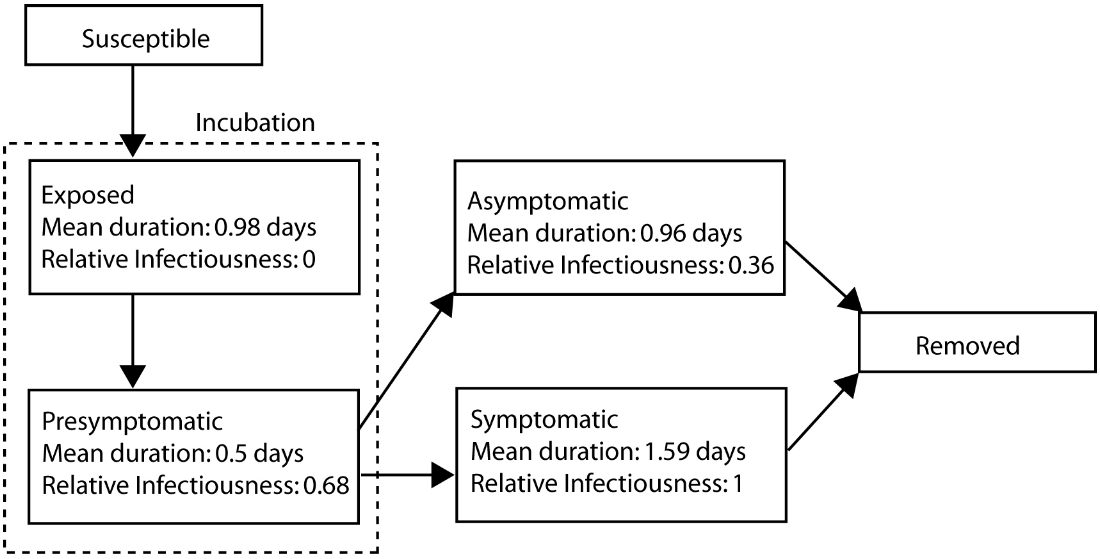

Figure A. Natural history of pandemic influenza. We have used this model previously to study mitigation strategies for pandemic influenza [4]. Individuals progressed from S (susceptible) through E (exposed but not infectious) to P (infectious and presymptomatic) to I (infectious and symptomatic, with probability $p_s = 0.67$) or A (infectious and asymptomatic, with probability $1 - p_s = 0.33$) and finally to removed. The value of p_S is consistent with recent modeling literature [3,13,14] and other sources: a basic reproductive number of 1.8 [15] with a 50% case attack rate, and observations from deliberate infections of humans with H1N1[16]. We used recent results [15] derived from a symptom-based household study [17] for the waiting time of the combined E and P stages: it is distributed according to an offset Weibull with offset +0.5 d, mean 1.48 d (including the offset), and standard deviation 0.47 d. The duration of the P stage was assumed to be fixed at 0.5 d. The duration of the I stage was set to be 5/3 that of the A stage, but the absolute duration of both stages was determined by the generation time, T_g = 2.6 days. The relative infectiousness for different disease stages shown were derived from more intuitive parameters. As in our previous study [4], we used θ (baseline value 0.3), the proportion of transmission occurred during P or A [18], rather than the relative infectiousness, as the primary parameter. See Ref 4 for the details on the parameter translation procedure. See Table A for the parameter baseline value and ranges for sensitivity analyses.

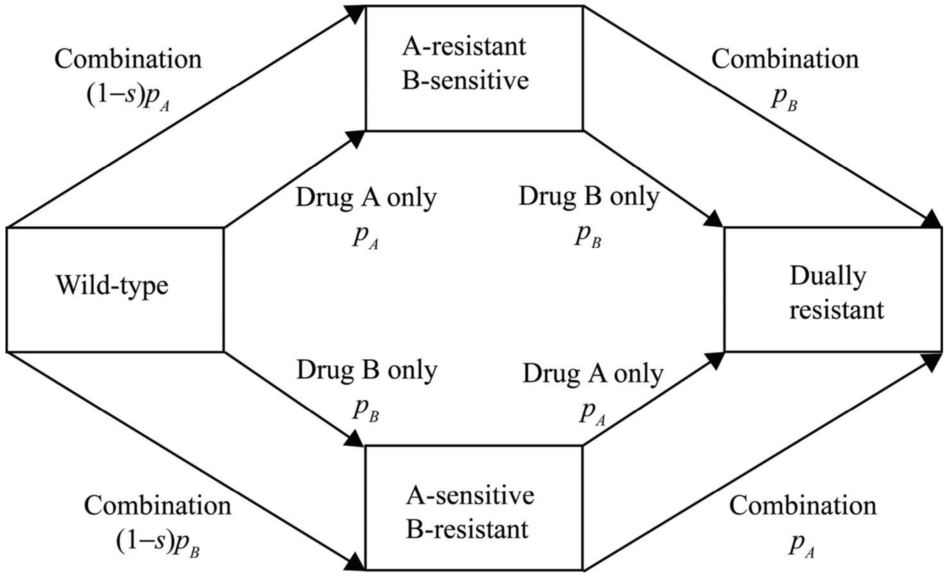

Figure B. The resistance emergence model. Each arrow represents a possible path for resistance emergence. A wild-type infection became dually-resistant in a step-wise fashion by first acquiring resistance to one drug and then to the other. However, emergence of resistance was a continuous-time process and a wild-type infection could become dually-resistant within a single individual. Rates of resistance emergence were assumed to be constant throughout the symptomatic stage. For drug $X \in \{A, B\}$, the rate of resistance emergence r_X was calculated from the probability of resistance emergence p_X via the formula $p_X = 1 - E\left[\exp(-r_X D_{Symptomatic})\right]$, where $E[\cdot]$ was the expectation operator and $D_{Symptomatic}$ was the symptomatic duration. We assumed that if an individual infected with strain w was treated with antiviral regimen d, his infectiousness was reduced by a proportion $\varepsilon = 0.66$ if strain w was sensitive to at least one drug in regimen d [1]. Although combination chemotherapy is likely to be more efficacious than monotherapy in reducing infectiousness [2], results were essentially the same when we set $\varepsilon = 1$ under combination chemotherapy (see *Reduction in infectiousness under combination chemotherapy*).

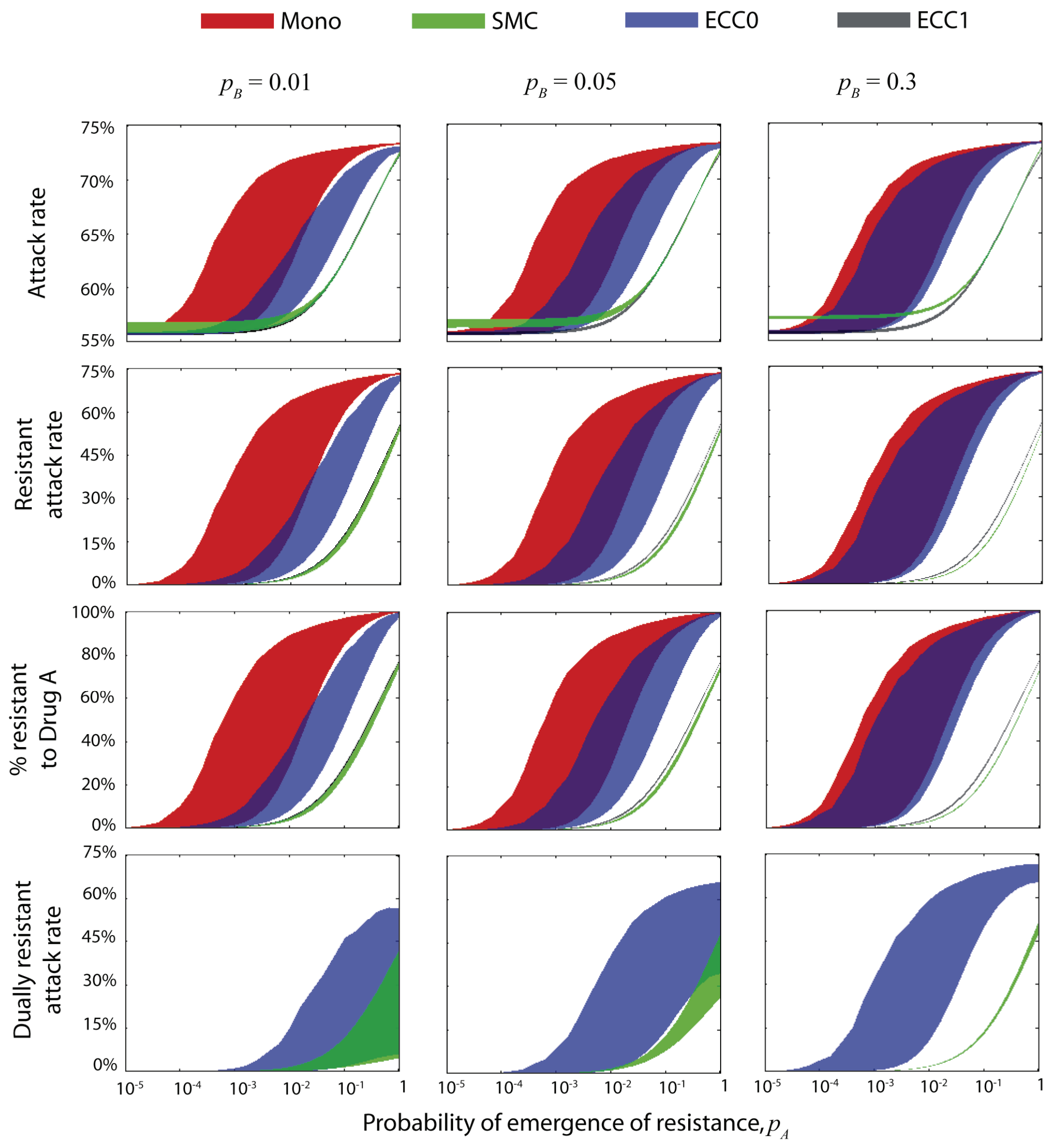

Figure C. Summary of the effectiveness of ECC and SMC for a closed population. Each column corresponds to a different value of p_B. Each row corresponds to a different outcome: attack rate (first row), resistant attack rate (second row), proportion of infections resistant to drug A (third), and number of infections resistant to both drugs A and B (fourth). ECC0 and ECC1 refer to ECC with $s = 0$ and $s = 1$, respectively.

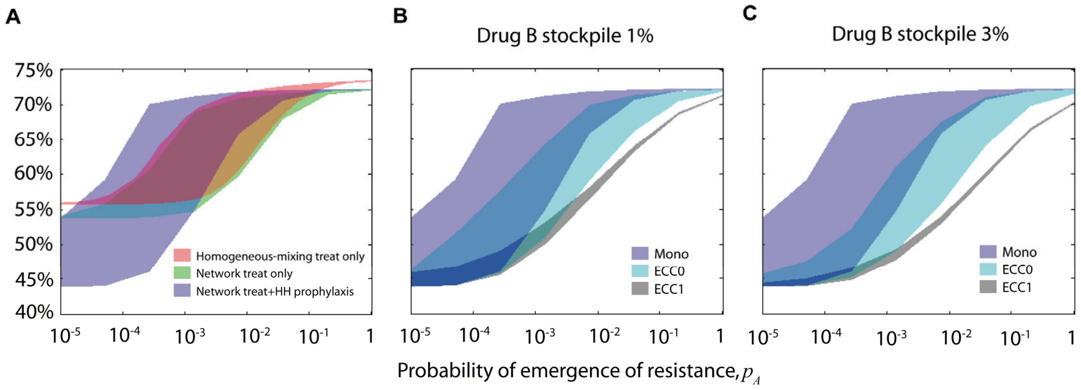

Figure D. Antiviral treatment plus household-based prophylaxis in a single population of 6.8 million with household- and workplace-structures ("the network model"). Shaded areas represent 95% prediction intervals. ECC0 and ECC1 refer to ECC with $s = 0$ and $s = 1$, respectively. Outcomes from the network model were estimated using 350 realizations. **A** The homogeneous mixing model (used in the main text) and the network model gave similar AR (and RAR) predictions when antiviral intervention involved only treatment of symptomatic individuals (red shade vs. green shade). **B** Effectiveness of ECC in reducing the spread of resistance in the presence of household-based antiviral prophylaxis with a drug B stockpile coverage of 1%. **C** Same as b but with a drug B stockpile coverage of 3%.

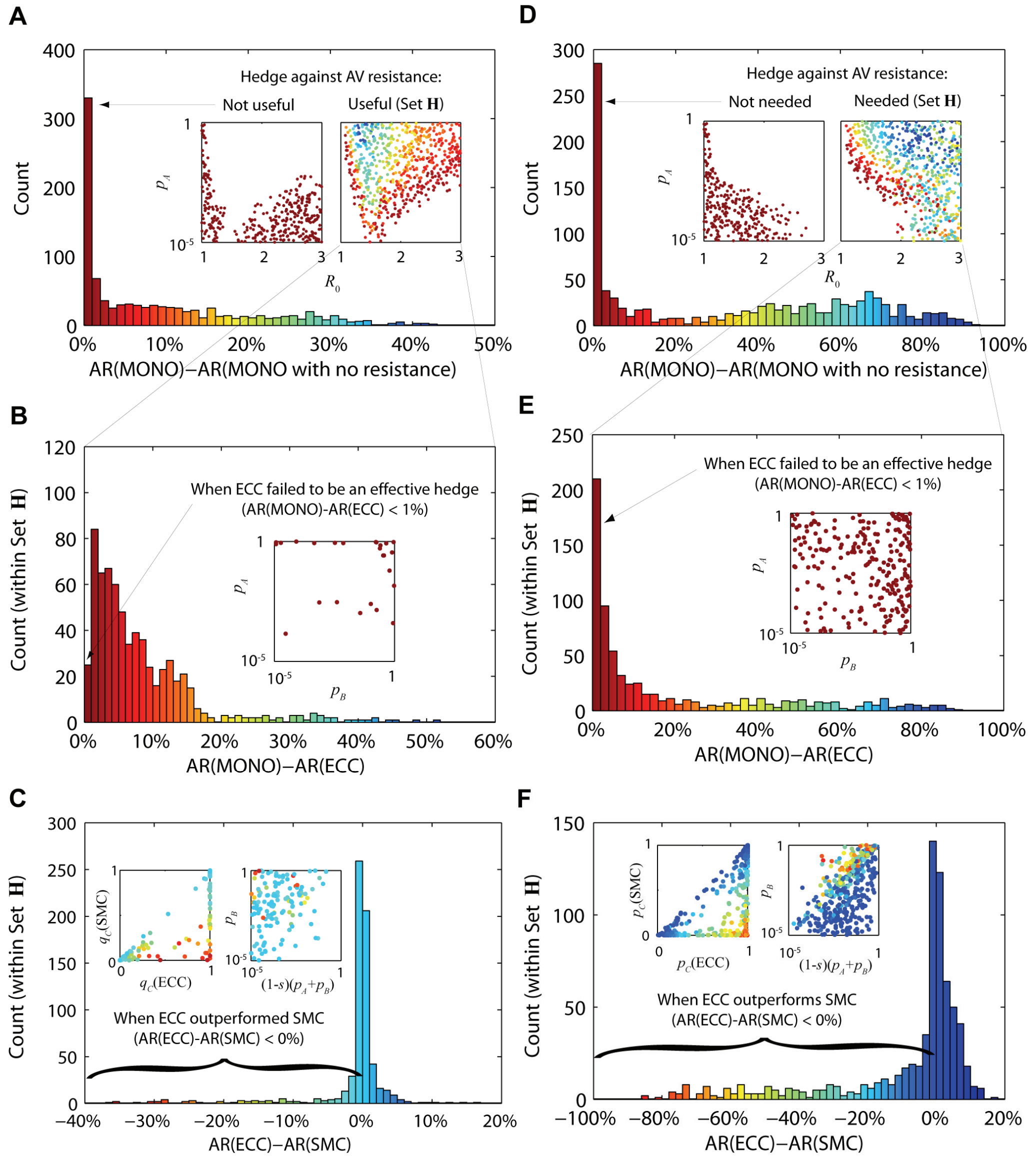

Figure E. Sensitivity analysis of the single-population simulation model. **A-C** This column is the same as Figure 2 except that the synergy parameter was chosen by randomly generating $1-s$ on log-scale between 10^{-7} and 1. ECC outperformed SMC (i.e. AR(ECC)–AR(SMC) < 0%) in 53% of the scenarios in part C, which was much higher than the 22% in Figure 2C. The reason is that most of the values of s generated were very high (72% and 86% of the s values generated were greater than 0.99 and 0.9. respectively). See Figure F for a more detailed examination of the effect of synergy on the relative performance of ECC and SMC. **D-F** This column is the same as Figure 2 except $p_T = 1$. ECC outperformed SMC (i.e. AR(ECC)–AR(SMC) < 0%) in 59% of the scenarios, which was much higher than the 22% in Figure 2C. The reason is that higher treatment probability tended to increase the proportion of scenarios where containment was likely under ECC but not under SMC.**”**

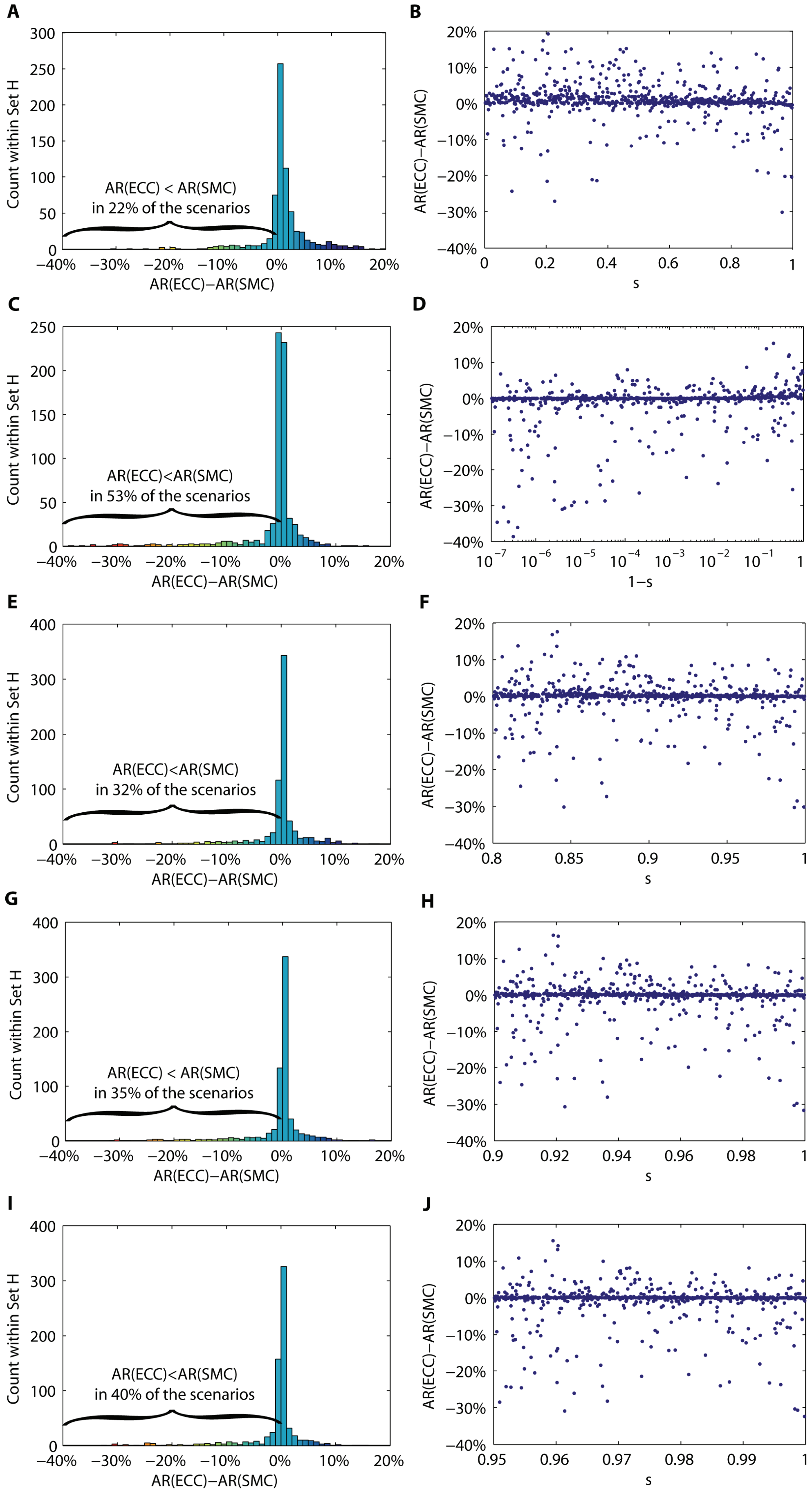
A
B
C
D
E
F
G
H
I
J
Count within Set H
AR(ECC)−AR(SMC)
AR(ECC) < AR(SMC)
in 22% of the scenarios
AR(ECC)<AR(SMC)
in 53% of the scenarios
AR(ECC)<AR(SMC)
in 32% of the scenarios
AR(ECC) < AR(SMC)
in 35% of the scenarios
AR(ECC)<AR(SMC)
in 40% of the scenarios
s
1−s

Figure F. A closer look at the performance of ECC and SMC in the single population sensitivity analysis. The transmission scenarios were the same as that in Figure 2 except that the synergy parameter s was chosen differently as follows: s chosen uniformly on a linear scale in [0,1] in **A-B** (i.e. as in Figure 2), [0.8,1] in **E-F**, [0.9,1] in **G-H**, and [0.95,1] in **I-J**; $1-s$ chosen uniformly on a log scale in $[10^{-7},1]$ in **C-D** (i.e. as in the left column of Figure E). Panels in the left column are analogous to Figure 2C, and parts C and F of Figure E. Panels in the right column show the sensitivity of AR(ECC)-AR(SMC) to the synergy parameter (note that on the x-axis, $1-s$ is plotted on log-scale in d while s is plotted on linear scale for the others). By comparing the proportion of scenarios for which ECC outperformed SMC in each row, we conclude that SMC is likely to outperform ECC unless synergy is very high (> 0.95).

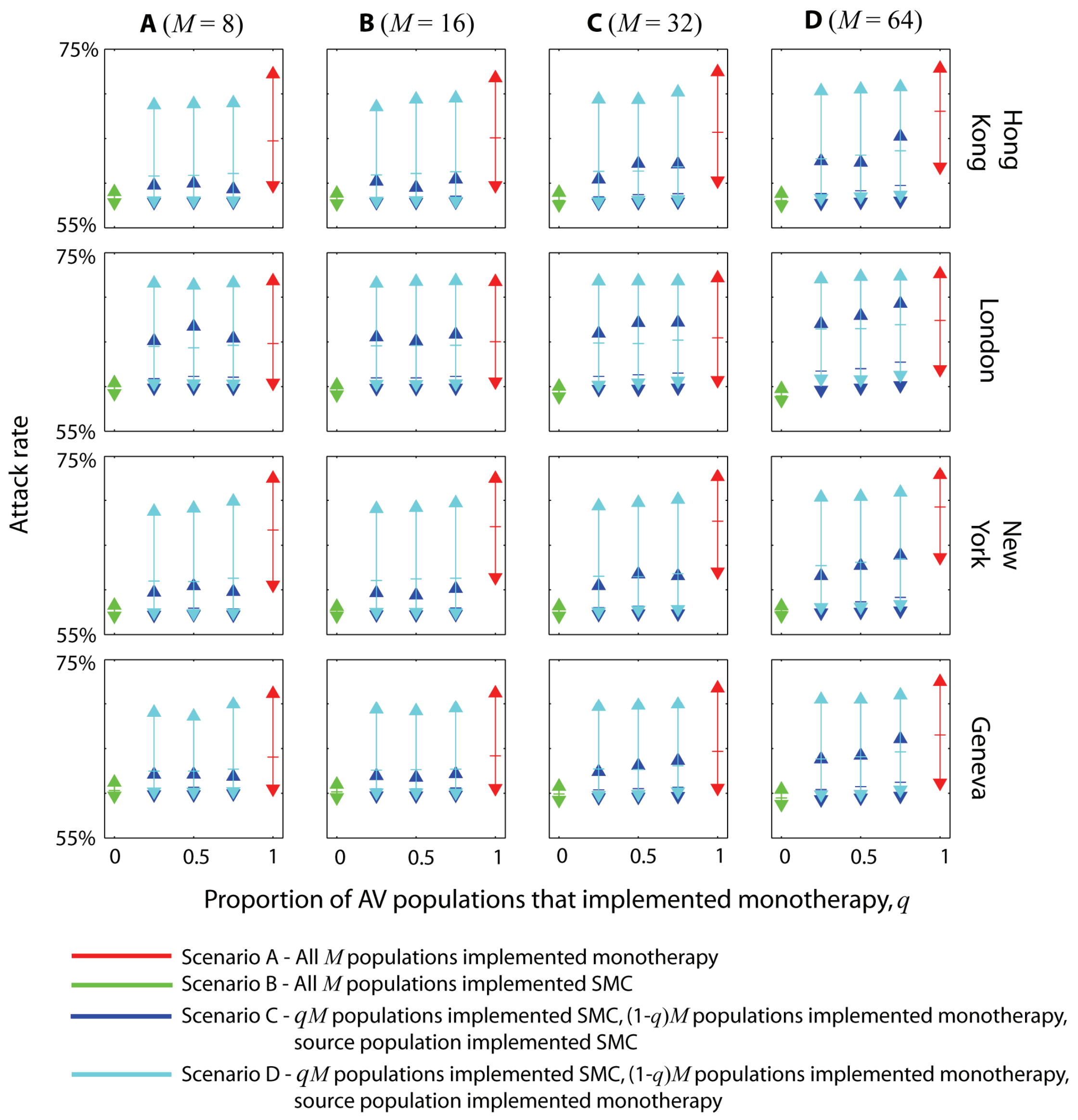

Figure G. Sensitivity of the effectiveness of SMC against the number of populations that implemented antiviral intervention (M) and monotherapy (qM). Vertical bars indicate 95% prediction intervals with triangles indicating the upper and lower bounds and horizontal dashes indicating the means (from 500 realizations). In each panel, the four colors correspond to the scenarios in Figure 3 (red – scenario A, green – scenario B, blue – scenario C, cyan – scenario D). Columns **A-C** correspond to different values of M. Each row corresponds to a different population (the same as in Figure 3).

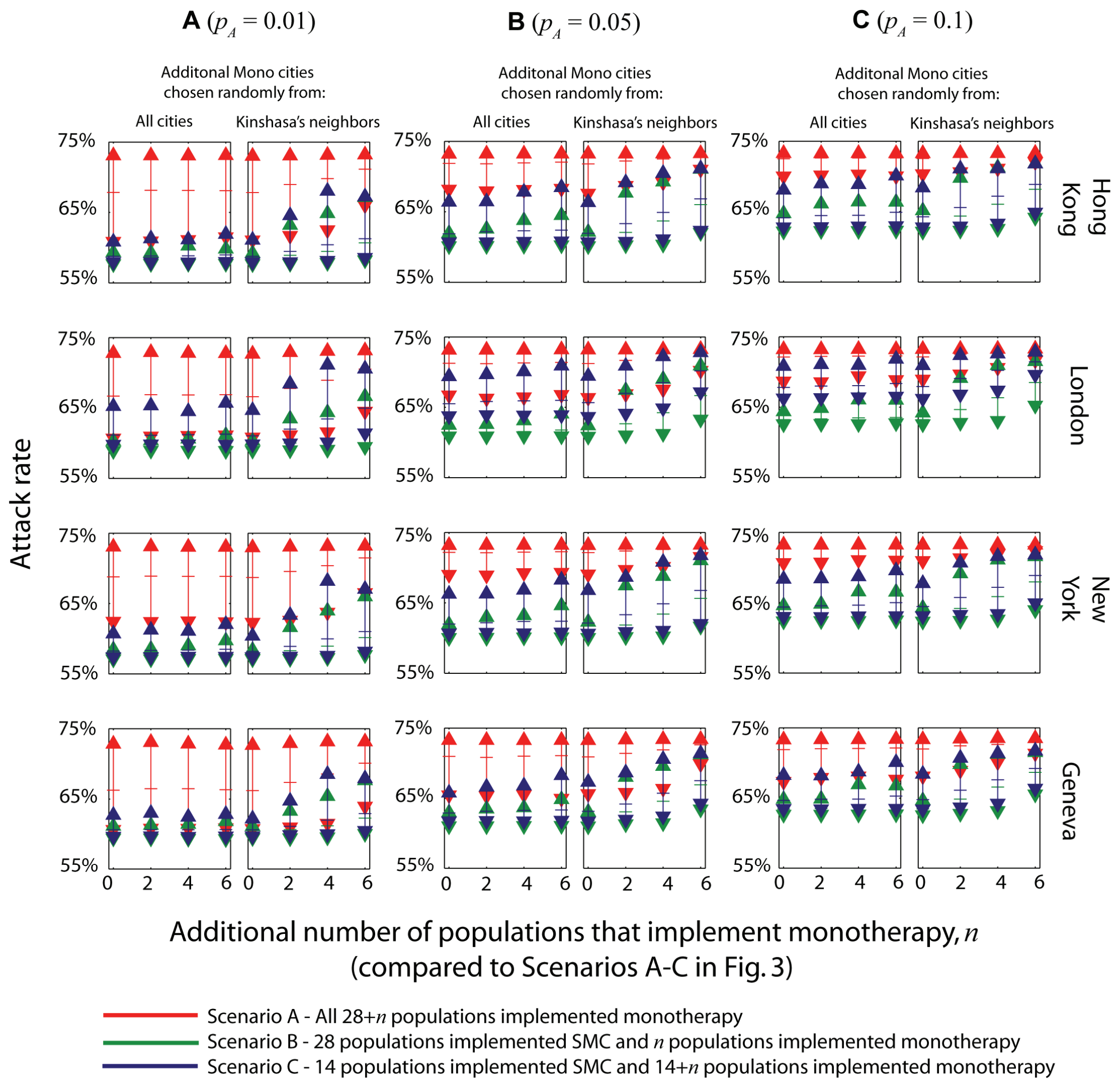

Figure H. An example to show how the effectiveness of SMC in downstream populations depends on the policy adopted by the neighbors of the source. See the subsection *When will SMC/ECC fail in downstream populations if the source does implement SMC/ECC* for detailed description. Vertical bars indicate 95% prediction intervals with triangles indicating the upper and lower bounds and horizontal dashes indicating the means (from 500 realizations). In each panel, the three colors correspond to the scenarios in Figure 3 (red – scenario A, green – scenario B, blue – scenario C). **A-C** Three values of p_A: 0.01, 0.05, 0.1. Each row corresponds to a different population (the same as in Figure 3).

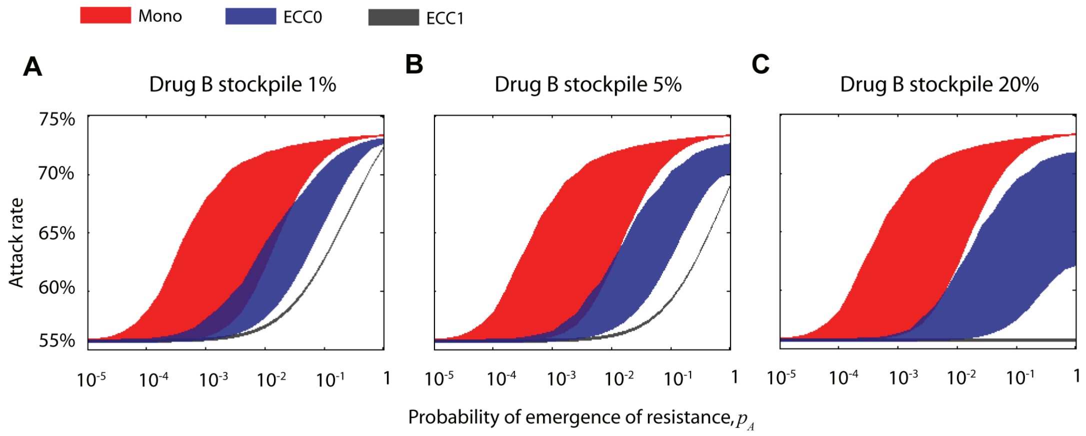

Figure I. The effectiveness of ECC for different stockpile sizes of drug B. ECC0 and ECC1 refer to ECC with $s = 0$ and $s = 1$, respectively. **A** 1% drug B coverage, as in the main text. **B** 5% drug B coverage. **C** 20% drug B coverage, which was sufficient to give combination chemotherapy to all treated cases throughout the entire epidemic.

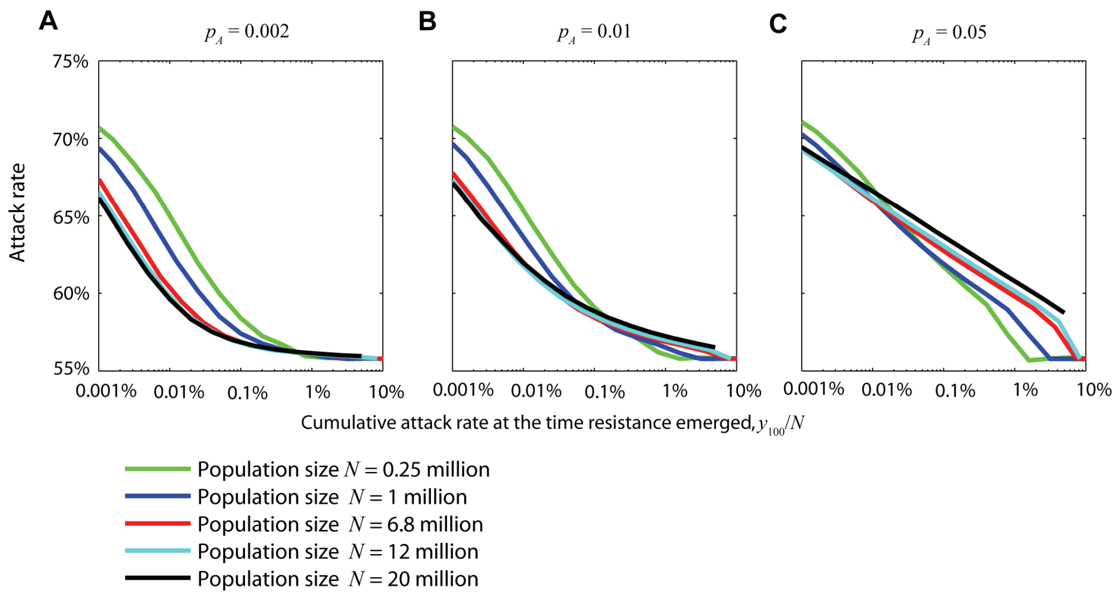

Figure J. The impact of delaying the emergence of resistance in populations of different sizes. Three values of probability of resistance emergence is considered: **A** $p_A = 0.002$; **B** $p_A = 0.01$; **C** $p_A = 0.05$. These figures are similar to Figure 1C except that the x-axes show the attack rate instead of the absolute number of infections. Five population sizes between 0.25 million and 20 million are considered in each panel. In the main text and Figure 1C, we argued that in a population of 6.8 million, the spread of resistance can be substantially reduced if the emergence of resistance can be delayed until the cumulative (wild-type) attack rate had reached 1.5% or above. This figure shows that the same conclusion applied for population sizes up to 20 million as long as the probability of emergence of resistance was within reasonable range (<0.05). Therefore, if 1% drug B coverage was sufficient for ECC and SMC to be effective in a population of 6.8 million (as in the main text), the same level of coverage was sufficient for all population sizes of interest in the global model. Consequently, in the global model, we assumed 1% drug B coverage for all populations that implemented SMC or ECC.

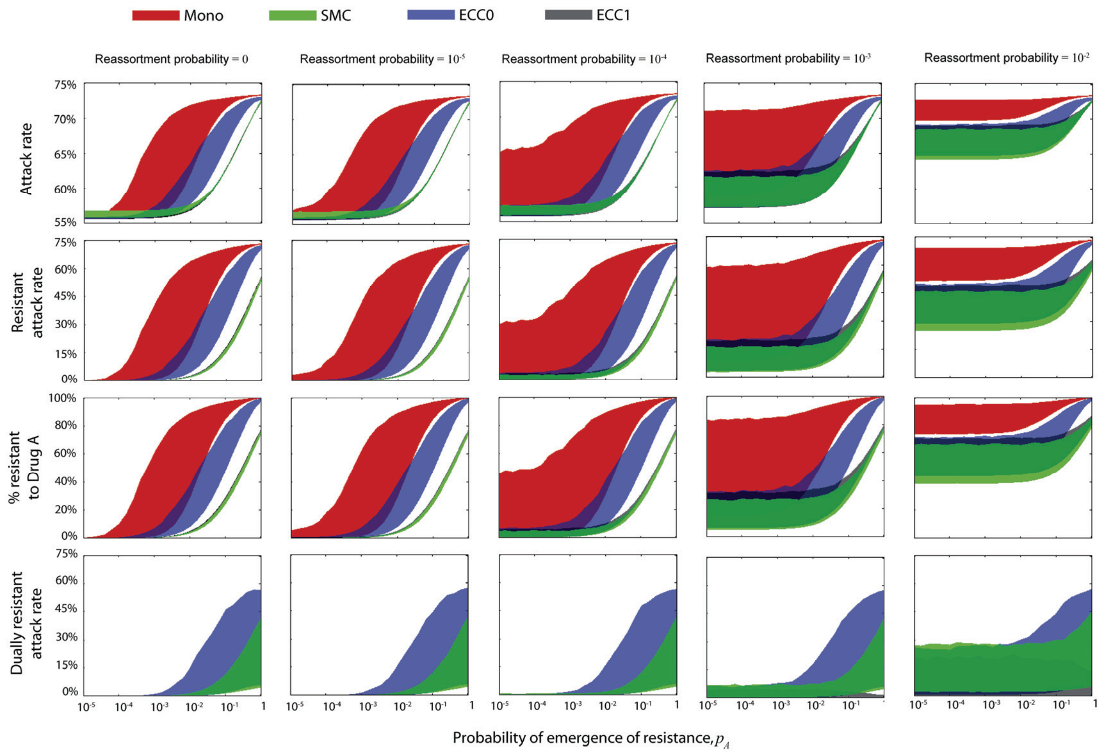

Figure K. The likely impact of resistance emergence via reassortment between the pandemic strain and circulating resistant seasonal strains. ECC0 and ECC1 refer to ECC with $s = 0$ and $s = 1$, respectively. Reassortment is crudely modeled by assuming that each pandemic infection has a certain probability v of becoming resistant to the primary antiviral.

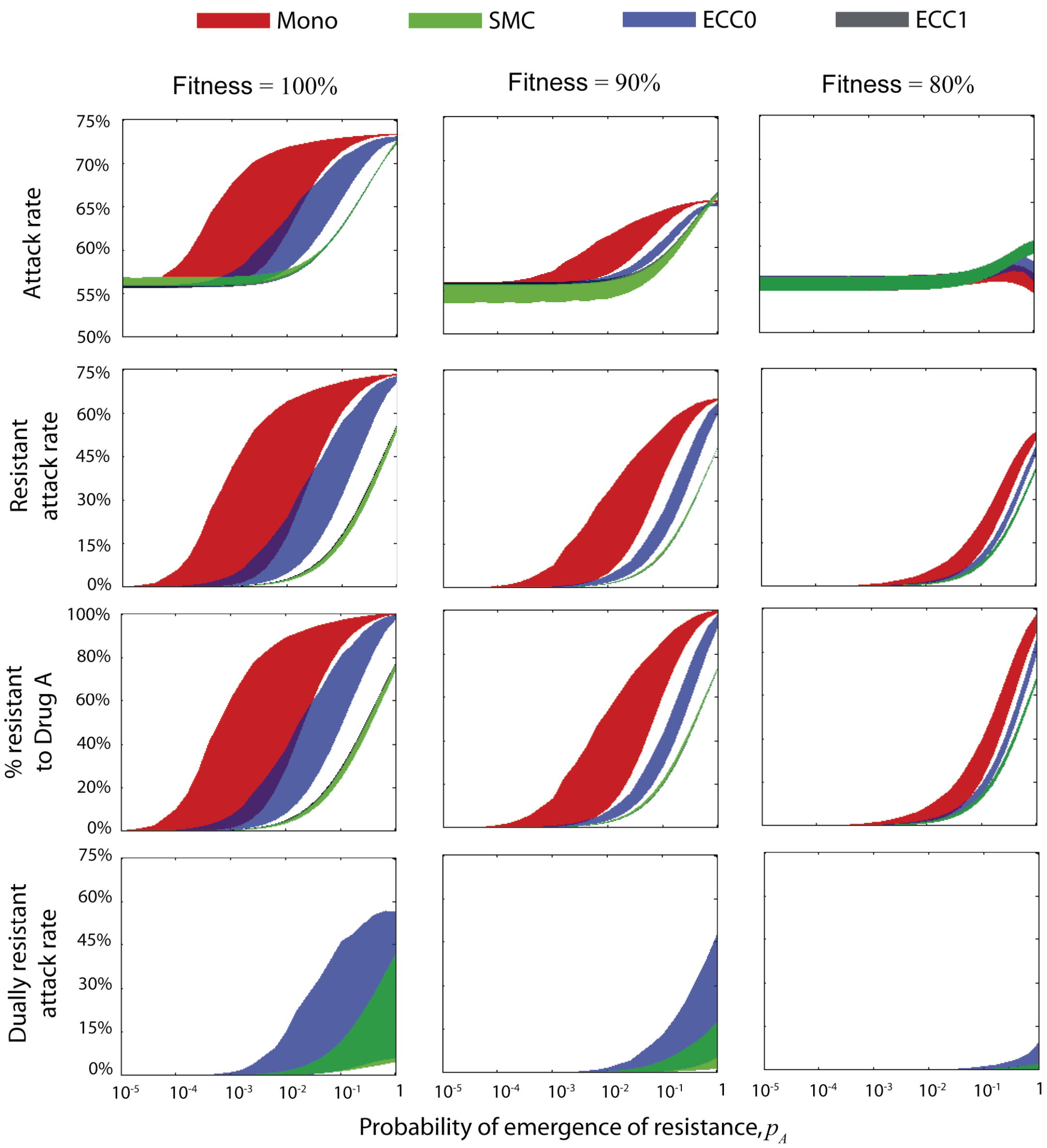

Figure L. The effect of fitness cost on the spread of antiviral resistance and effectiveness of SMC and ECC. Each column corresponds to a different value of fitness cost. Each row corresponds to a different outcome: attack rate (first row), resistant attack rate (second row), proportion of infections resistant to drug A (third), and number of infections resistant to both drugs A and B (fourth). ECC0 and ECC1 refer to ECC with *s* = 0 and *s* = 1, respectively.

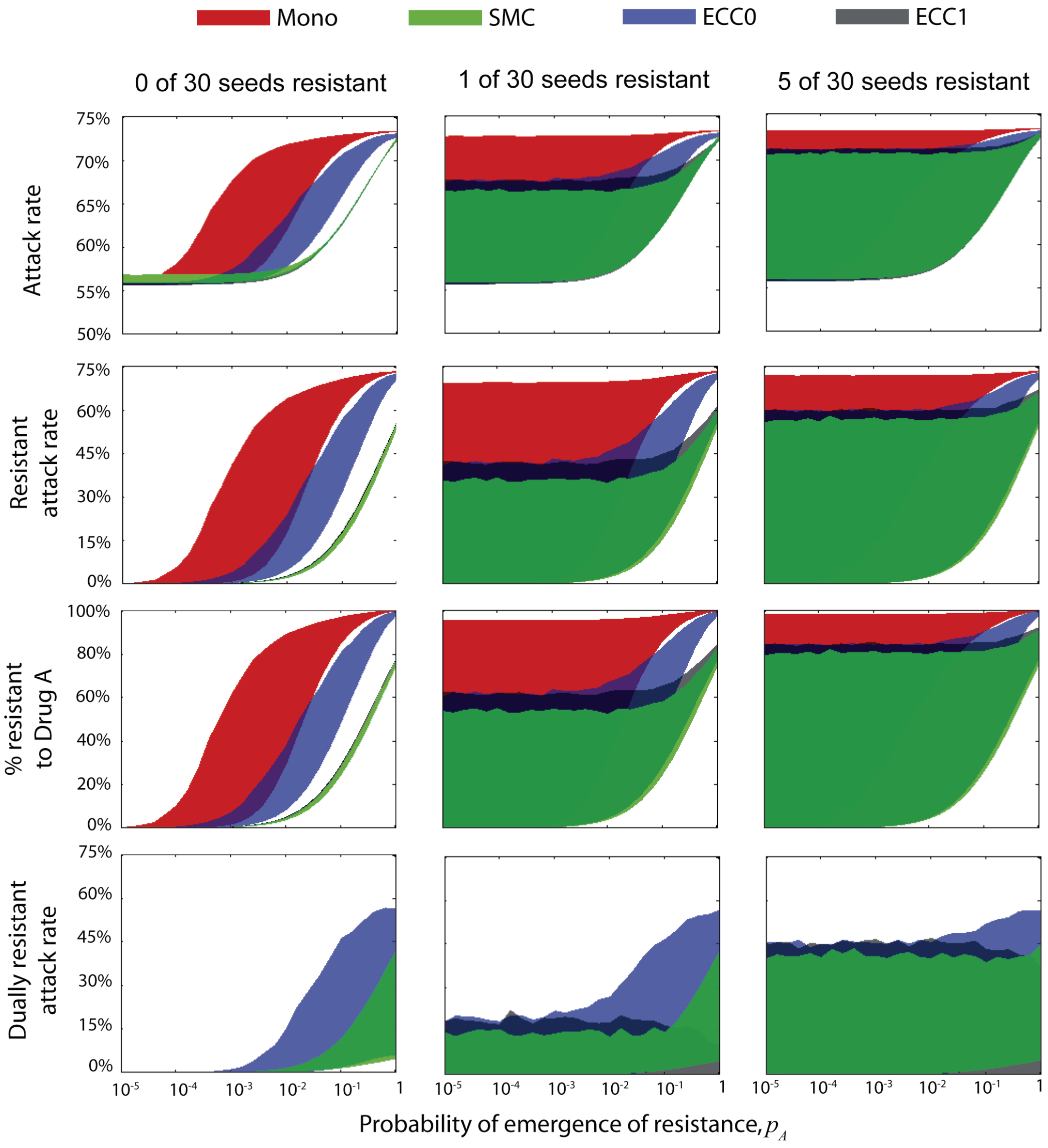

Figure M. The effect of *de novo* resistance on the spread of antiviral resistance and effectiveness of SMC and ECC. Each column corresponds to a different number of resistant seeds at the beginning of the epidemic. Each row corresponds to a different outcome: attack rate (first row), resistant attack rate (second row), proportion of infections resistant to drug A (third), and number of infections resistant to both drugs A and B (fourth). ECC0 and ECC1 refer to ECC with s = 0 and s = 1, respectively.

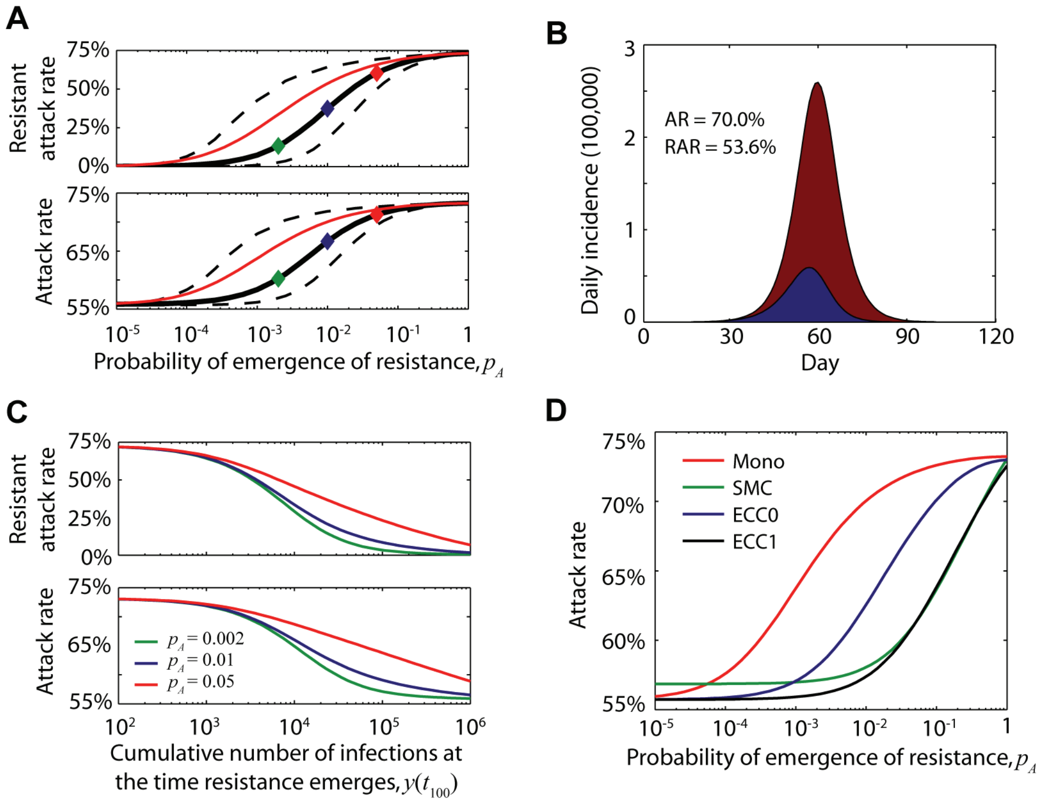

Figure N. Dynamics of resistance emergence and mitigation in a single population using the deterministic model. This figure is analogous to Figure 1 in the main text. **A** This chart is the same as Figure 1A with the outcomes in the deterministic model shown by the red curves. **B** The single-population epidemic in the deterministic model with $p_A = 0.01$ (brown shaded area corresponds to resistant incidence; blue shaded area corresponds to wild-type incidence; AR, attack rate; RAR, resistance attack rate). **C** AR and RAR as functions of the cumulative number of wild-type infections at the time resistance emerged (colors correspond to the value of p_A as per diamonds in part a; in the absence of interventions AR=73% and RAR=0%; in the absence of resistance, AR=56% and RAR=0%). **D** Efficacy of early combination chemotherapy (ECC, antiviral synergies of $s = 0$ and $s = 1$) and sequential multi-drug chemotherapy (SMC) in reducing the attack rate.
